# When seeing stigma creates paternalism: Learning about disadvantage leads to perceptions of incompetence

**DOI:** 10.1177/13684302211009590

**Published:** 2021-05-29

**Authors:** Stephanie L. Reeves, Crystal Tse, Christine Logel, Steven J. Spencer

**Affiliations:** 1 Fors Marsh Group, Arlington, VA, USA; 2 Center for the Advancement of Teaching Excellence, University of Illinois at Chicago Academic Center for Excellence, University of Illinois at Chicago, USA; 3 Department of Social Development Studies, Renison University College, Affiliated with University of Waterloo, Waterloo, ON, Canada; 4 Department of Psychology, The Ohio State University, Columbus, OH, USA

**Keywords:** disadvantage, intergroup relations, paternalism, prejudice, stigma

## Abstract

The present research examines the conditions under which educating non-stigmatized individuals about the experiences of members of stigmatized groups leads to paternalistic or more respectful views of the target. We propose that when these efforts ask members of non-stigmatized groups to focus only on the difficulties experienced by stigmatized targets, they will lead to more paternalistic views of targets because they portray targets as being in need of help. In contrast, we propose that when these efforts take a broader focus on stigmatized targets and include their resilience in the face of their difficulties, they will lead to more respectful views of targets. Four studies supported these predictions. Across studies, White participants who focused only on a Black target’s difficulties subsequently perceived the target as more helpless and less competent than controls. Participants who focused on the target’s resilience in the face of difficulties perceived him as more competent.

Group-based inequities are an unfortunate reality in modern society. Whereas members of non-stigmatized or majority groups (e.g., White, upper class, straight, etc.) are afforded privileges and benefits from their group membership, members of stigmatized groups often face considerable disadvantages and marginalization. In the context of such marginalization, intergroup interactions can be fraught with misunderstanding, tension, and ultimately less satisfaction for interaction partners (Bergsieker et al., 2010).

In recent years, some have argued (Adams et al., 2007; Goodman, 2001; Katz, 2003) that one way to improve intergroup relations is to educate non-stigmatized individuals about the experiences of stigmatized groups. But do these efforts necessarily lead to less prejudice and discrimination? Without denying their value overall, we argue that when such efforts focus only on the difficulties that stigmatized individuals experience, they may in fact backfire by leading to more negative perceptions of stigmatized groups.

To illustrate why this might be the case, consider as an example a White, middle-class teacher who is attempting to understand the experiences of a struggling student; the student’s grades are lower than they could be, but she also knows that this student comes from a working-class, low-income family and that there are negative stereotypes characterizing his group as intellectually inferior. One way that this teacher could approach the student would be to think about how difficult the student’s plight must be – about how she might feel if she were faced with such hardship. Feeling sorry for the student, the teacher might lower her standards for him: assigning easier problem sets, or calling on him fewer times in class. Although this strategy may lead the teacher to feel sympathy for the student, and perhaps even to like the student more, it does not allow the teacher to appreciate his strengths. By lowering the teacher’s expectations for the student, this approach to understanding the student’s experiences could ultimately prevent him from fulfilling his full academic potential.

An alternative way the teacher could attempt to understand the student’s experiences would be to focus on the student’s strengths and resilience in the face of the barriers he faces. With a newfound appreciation of the student and his strengths, the teacher may choose to provide support and encouragement that would allow the student to improve without lowering her standards for him. Although the differences between these two ways of approaching stigma may seem subtle, they could lead to very different outcomes for both the teacher and the student. Following from these possibilities, in the present research we propose two different ways of thinking about societal stigmatization and suggest that these two approaches will have different consequences for perceptions of stigmatized groups.

Although there are many ways that one can think about stigma, we propose an important distinction between two such ways in particular. The first we refer to as a *difficulties-focused approach*, which is characterized by a narrow focus on the struggles and difficulties that someone experiences as a result of stigma. We hypothesize that this approach will cause people to view targets as helpless victims, rather than as competent individuals deserving of respect. In contrast, one could take what we refer to as a *resilience-focused approach* to stigma, which is characterized by a focus on a person’s resilience in the face of their difficulties. We suggest that this way of thinking about stigma will lead to increased appreciation of targets’ competencies, and ultimately increased respect for targets.

## Normative Models of Person Perception

The notion that learning about another person’s experiences of stigma could undermine perceptions of their competence stands in contrast to normative models of person perception (Jones & Davis, 1965; Kelley, 1973). Indeed, according to the *augmentation principle*, learning that another person has acted in the face of a constraint should lead people to make stronger inferences about the internal causes of the action than if the constraint were not present (Kelley, 1973). For example, if we learn that an individual has graduated from college despite being from a group that has been historically stigmatized and excluded in college, we should infer that the individual is a *stronger* student than someone who graduated from college but did not contend with such marginalization. Similarly, if a student struggles in college because they are stigmatized and excluded based on their group membership, people should recognize that the student has acted in the face of a constraint and adjust their perception of the student’s competence accordingly. Thus, a stigmatized student who struggles in college should be perceived as a stronger student than a non-stigmatized student who struggles. The augmentation principle therefore predicts that focusing on the difficulties that a member of a stigmatized group faces should lead non-stigmatized group members to perceive that individual as relatively *more* competent.

Empirical evidence, however, has shown that people often fail to take into account constraining forces when making causal attributions for another person’s behavior (Jones & Harris, 1967; Ross, 1977; Ross et al., 1977). Indeed, hundreds of studies have shown that people tend to give precedence to internal (or person-based) explanations for behavior over external ones, a phenomenon known as the fundamental attribution error (for a review, see Malle, 2011). For instance, in one classic study, people read essays that were either for or against Fidel Castro (Jones & Harris, 1967). People rated writers of pro-Castro essays as having relatively more positive attitudes toward Castro, even when they knew that the writer’s positions on the essay were determined by a coin toss. With respect to learning about others’ experiences of stigma, the fundamental attribution error predicts that people should fail to take into account the impact of such experiences when perceiving the actions of stigmatized individuals. Returning to the previous example, for instance, people would see the two college graduates as equally strong students even though one has graduated in spite of group-based stigmatization that constrains academic performance.

## Paternalistic Prejudice

Whereas attribution theory predicts that learning about stigma should be *ineffective* at improving non-stigmatized group members’ perceptions of stigmatized groups, we suggest that doing so may even *undermine* perceptions to the extent that it creates a sense of *paternalism* (Fiske et al., 2002). Previous research has defined paternalistic prejudices as those that involve seeing the target as someone who is not competent, and who needs to be protected and cared for (Glick & Fiske, 1996). For instance, gender prejudice can often take a paternalistic form, in which women are viewed positively, but also as fragile, incompetent, and in need of protection. Why might focusing on the difficulties that a stigmatized individual faces lead to paternalism? We suggest that it may do so because it portrays the target as someone who needs help. Previous research has shown that people who receive help are often seen as less competent than those who do not receive help. In one study, Gilbert and Silvera (1996) found that third-party observers saw people who received many hints on an anagram task (i.e., those who were “overhelped”) as less competent than people who received few hints on an anagram task (i.e., those who were “underhelped”). Other research has suggested that receiving help can also cause recipients to doubt their *own* competence. In one study, African American students who received unsolicited help from a White student subsequently doubted their competence more than those who did not receive help (Schneider et al., 1996).

Based on this reasoning, we suggest that learning about someone’s experiences with stigmatization may lead to paternalistic views of that individual to the extent that it leads people to see that person as in need of help. People may genuinely feel bad about the target’s plight, but by seeing them as being in such need of help, they will also likely view them as less competent. Thus, we expect that when people learn about the experiences of stigmatized groups while focusing *only on their difficulties*, they will see them as more in need of help and as less competent. In short, they will be viewed paternalistically.

Is such a reaction inevitable whenever a non-stigmatized group member learns about stigma? Our reasoning suggests that paternalism is not a necessary consequence of such efforts. Perhaps counterintuitively, we reason that the problem with this approach to stigma is that it focuses narrowly on people’s difficulties, rather than viewing them as complex individuals with difficulties but also strengths and resiliencies. If efforts to educate people about stigma instead focus more broadly on a stigmatized target and include their *resilience in the face of their difficulties*, we suggest that they will lead people to respect the target and to appreciate their competence.

In light of these possibilities, the present research examines the effects of these two different approaches to stigma on perceptions of stigmatized targets. We hypothesize that focusing only on the difficulties that a stigmatized individual faces will lead non-stigmatized group members to view the target as more helpless and less competent – that is, it will lead them to view the target paternalistically. In contrast, we hypothesize that focusing not only on a stigmatized target’s difficulties but also on their resilience in the face of these difficulties will lead people to view the target as more competent.

## The Present Research

In the present research, we examined whether the two ways of approaching stigma – the difficulties-focused approach vs. the resilience-focused approach – might have different consequences for perceptions of stigmatized individuals. We examined this question in an important applied context: empathy interventions to improve Whites’ perceptions of Black people. Empathy interventions to reduce prejudice towards socially stigmatized groups are an area in which the distinction between the difficulties-focused approach and the resilience-focused approach to stigma should be particularly relevant. Indeed, such interventions often take the form of asking a non-stigmatized individual to imagine a stigmatized individual’s experiences with the difficulties associated with belonging to a socially stigmatized group, such as being the target of prejudice and discrimination or contending with negative stereotypes (Batson et al., 2002; Batson et al., 1997; Vescio et al., 2003). The assumption behind these interventions is that taking the perspective of a member of a stigmatized group as they experience hardship and struggle will lead to more care and concern about that person’s welfare, which will then translate to more positive attitudes toward the stigmatized group as a whole (Batson et al., 1997). According to our reasoning, however, such interventions may backfire (i.e., by leading people to view the targets paternalistically) to the extent that they ask people to focus exclusively on the difficulties that a stigmatized individual faces. If, on the other hand, empathy interventions asked people to focus on stigmatized individuals’ resilience in the face of their difficulties, they could have beneficial outcomes for intergroup relations.

We tested our hypotheses in a series of five studies. Study 1 was an initial test of the effects of these two different approaches to stigma. Specifically, in Study 1, White participants were randomly assigned to complete an empathy intervention in which they took one of the two approaches described above, or a control approach, to a Black student’s experiences and then reported their impressions of the student. Study 2 sought to replicate the effects of Study 1 using a cleaner and more simplified version of the manipulation. Study 3 sought to rule out differences in information provided about the target across conditions as an alternative explanation of the effects. Specifically, in Study 3 we presented all participants with the same information about a Black target, and manipulated their construals of the target’s experiences such that they would interpret them in light of the target’s difficulties or their resilience. In Study 4, we examined whether empathy is a necessary component in the effects of the two approaches to stigma. To do so, we randomly assigned participants to empathize with the target or to take an objective perspective towards the target and crossed this manipulation with a manipulation of approach to stigma. Finally, Study 5 examined whether the effects would occur outside of the context of an empathy intervention or manipulation. In this study, participants were simply asked to read about the target’s experiences while focusing on their difficulties, their resilience, or neutral experiences; they received no instructions about whether to empathize with the target or to take a neutral perspective towards the target. All measures, manipulations, and exclusions are disclosed here and in the online supplemental materials.^1^

## Study 1

The goal of Study 1 was to provide an initial test of whether an empathy intervention that focused on a stigmatized target’s difficulties vs. their resilience in the face of their difficulties would have different implications for White individuals’ perceptions of the target. To do so, we first asked participants to empathize with a Black target while focusing on the target’s difficulties, their resilience in the face of their difficulties, or neutral experiences. We then examined the extent to which they viewed the target as helpless, as well as their perceptions of the target’s competence. We predicted that focusing on the Black target’s difficulties would lead White participants to view the target as more helpless and less competent, whereas focusing on the target’s resilience would lead participants to view the target as more competent.

### Method

#### Participants

One hundred forty-six White American Mechanical Turk workers (51.4% male, 48.6% female; *M*_age_ = 33.4 years, *SD* = 12.2) participated in the study in exchange for payment.^2^ We conducted a sensitivity power analysis with G*Power (Faul et al., 2007) to determine the smallest effect size we could have detected with this sample size for our focal contrasts. This analysis revealed that we had 80% power to detect an effect size of *d* = 0.58 for both control condition vs. experimental condition contrasts, and an effect size of *d* = 0.57 for the difficulties condition vs. resilience condition contrast with an independent samples *t*-test at *p* < .05.

#### Materials and Procedure

Participants completed all materials and procedures online. They were told that the researchers were pilot testing reading and writing materials, and that they would read and write about randomly chosen topics. Participants were then randomly assigned to complete a focus manipulation in which they learned about someone experiencing stereotype threat (i.e., one form of stigmatization; Steele, 1997) and focused on either this person’s difficulties, their resilience, or on neutral aspects of this person’s experiences. After the focus manipulation, participants completed a series of self-report dependent measures.

#### Focus Manipulation

The focus manipulation consisted of reading a brief article and then completing a perspective-taking writing task. The content of the article and perspective-taking activities differed based on condition. Participants in the two experimental conditions (i.e., the difficulties-focus condition and the resilience-focus condition) first read an article about stereotype threat (Steele, 1997). The article guided readers through a thought experiment of experiencing stereotype threat, then taught them about stereotype threat and its effects on people with negatively stereotyped identities. Importantly, the content of the stereotype threat article did not differ across the two experimental conditions. We included this article in both conditions because the perspective-taking activity focused on a negatively stereotyped target’s experiences contending with stereotype threat. We thought it may be necessary for participants to first learn about stereotype threat to fully understand and appreciate the perspective-taking activity. Thus, the purpose of this article was to give participants in both experimental conditions a basic overview of what experiencing stereotype threat entails to provide context for the next part of the manipulation. Participants in the control condition read a neutral article about plants.

After reading their respective articles, participants completed a perspective-taking exercise that was modeled after empathy interventions used in previous research (Batson et al., 1997). In all three conditions, participants were instructed to write a brief summary about a day in the life of Tyrone Williams, who was ostensibly a Black college student. Consistent with past research (Batson et al., 1997), they were instructed to “try to feel the full impact of his experiences, and how he feels as a result.” Participants in the *difficulties-focus condition* were further instructed to “try to imagine how much more difficult his experiences are than the typical student, and the kind of help he might need in such situations”; whereas participants in the *resilience-focus condition* were instructed to “try to imagine how much stronger he will become from these difficult experiences, and what he could teach you about dealing with challenging situations.”^3^ Participants in the control condition received no such further instructions.

All participants were then asked to write one sentence about how they might feel if they were Tyrone in five different scenarios. In the difficulties condition, these scenarios focused on the struggles and difficulties that Tyrone faces as a result of being a member of a negatively stereotyped group. In the resilience condition, the scenarios focused on Tyrone’s resilience in the face of his difficulties. For instance, one scenario in the difficulties condition asked participants to write about how Tyrone might feel when he “meets with his English professor to talk about his term paper, and how he might worry that the professor might judge his abilities in the light of negative stereotypes about Black people”; whereas in the resilience condition they were asked to write about how he might feel when he “meets with his English professor to talk about his term paper, and what strategies he uses to get the most out of the feedback despite the professor’s potential doubts about his ability.” The control condition included scenarios that were identical to those in the experimental conditions, but without the focus on difficulties or resilience. For instance, the analogous control scenario was: “meets with his English professor to talk about his term paper.”

#### Measures

The primary dependent measures consisted of participants’ perceptions of the extent to which the target possessed traits related to helplessness and competence. Each trait was rated on a five-point scale (1 = not at all; 5 = extremely). Two composites were formed by averaging participants’ ratings of the traits related to helplessness (*victim, incapable, in need of help, weak, needs support*; α = .77), and those related to competence (*empowered, strong, intelligent, determined, able to help others, has a lot to offer*; α = .84).^4^ Although these measures did not come from a previously validated scale, they showed reasonable internal consistency and provided a face valid measurement of helplessness and competence, respectively.

### Results

Data and syntax can be accessed by contacting the corresponding author. Table 1 displays means and standard deviations for outcomes for each study.

**Table 1.** Means and standard deviations.

Study	Outcome	Focus condition		
		Control	Difficulties	Resilience
Study 1	Helplessness	*M* = 2.10	*M* = 2.58	*M* = 2.21
		(*SD* = 0.63)	(*SD* = 0.63)	(*SD* = 0.71)
	Competence	*M* = 3.89	*M* = 3.64	*M* = 4.11
		(*SD* = 0.53)	(*SD* = 0.57)	(*SD* = 0.63)
Study 2	Helplessness	*M* = 1.94	*M* = 2.61	*M* = 2.05
		(*SD* = 0.70)	(*SD* = 0.80)	(*SD* = 0.68)
	Competence	*M* = 3.92	*M* = 3.66	*M* = 4.18
		(*SD* = 0.59)	(*SD* = 0.78)	(*SD* = 0.55)
Study 3	Helplessness	–	*M* = 2.27	*M* = 4.00
		–	(*SD* = 0.67)	(*SD* = 0.67)
	Competence	–	*M* = 3.77	*M* = 4.00
		–	(*SD* = 0.67)	(*SD* = 0.67)
Study 4	Helplessness	*M* = 1.96	*M* = 2.45	*M* = 2.07
		(*SD* = 0.56)	(*SD* = 0.60)	(*SD* = 0.54)
	Competence	*M* = 3.72	*M* = 3.56	*M* = 3.92
		(*SD* = 0.60)	(*SD* = 0.66)	(*SD* = 0.57)
Study 5	Helplessness	*M* = 1.68	*M* = 2.48	*M* = 1.89
		(*SD* = 0.43)	(*SD* = 0.65)	(*SD* = 0.52)
	Competence	*M* = 3.84	M = 3.62	*M* = 4.07
		(*SD* = 0.54)	(SD = 0.71)	(*SD* = 0.61)
	Admiration	*M* = 3.48	*M* = 3.39	*M* = 3.68
		(*SD* = 0.80)	(*SD* = 0.89)	(*SD* = 0.72)
	Affiliation	*M* = 3.48	*M* = 3.45	*M* = 3.78
		(*SD* = 0.81)	(*SD* = 1.04)	(*SD* = 0.86)

*Note.* Study 4 also included a manipulation check of an empathy manipulation that was not included in other studies, as reported in the main text and supplement. Means and *SD*s for the two Study 4 outcome variables reported here collapse across the empathy manipulation, but simple comparisons for the whole model are reported in the main text.

#### Helplessness

Consistent with our predictions, focus condition affected ratings of helplessness, *F*(2, 143) = 6.99, *p* = .001. Participants in the difficulties condition (*M* = 2.58, *SD* = 0.63) rated the target as more helpless than did participants in the control condition (*M* = 2.10, *SD* = 0.63; *t*[143] = 3.56, *p* = .001, *d* = 0.76) and the resilience condition (*M* = 2.21, *SD* = 0.71; *t*(143) = 2.75, *p* = .007, *d* = 0.55). In contrast, participants in the resilience condition viewed the target as no more helpless than did those in the control condition, *t*(143) = 0.86, *p* = .39, *d* = 0.16.

#### Competence

Focus condition also influenced ratings of competence, *F*(2, 143) = 8.16, *p* < .001. Participants in the difficulties condition (*M* = 3.64, *SD* = 0.57) rated the target as less competent than did those in the control condition (*M* = 3.89, *SD* = 0.53; *t*[143] = 2.14, *p* = .034, *d* = 0.45) and those in the resilience condition (*M* = 4.11, *SD* = 0.63; *t*[143] = 4.04, *p* < .001, *d* = 0.78). Participants in the resilience condition rated the target as marginally more competent than did those in the control condition, *t*(143) = 1.81, *p* = .072, *d* = 0.38.

### Discussion

These findings provide preliminary evidence that the two ways of approaching stigma (i.e. the difficulties-focused approach vs. the resilience-focused approach) have different consequences for Whites’ perceptions of stigmatized groups. Indeed, participants who focused only on the target’s difficulties and struggles perceived him as more helpless and less competent than controls. In contrast, participants who focused on the target’s resilience perceived him as more competent than controls.

A limitation of Study 1, however, is that participants in the experimental conditions read an article about stereotype threat prior to the perspective-taking activity. It is therefore unclear whether learning about stereotype threat is necessary for the observed effects to emerge, or if the differences between the two focus conditions would emerge with the perspective-taking exercise alone. Thus, in Study 2 we removed the stereotype threat article from the focus manipulation to provide a more straightforward test of our model.

## Study 2

In Study 2, we sought to replicate the primary findings of the previous studies using a cleaner manipulation (i.e., one that did not include the stereotype threat article). In addition, we pre-registered our hypotheses and analysis plan prior to collection of the data on the Open Science Framework (Open Science Framework, n.d.; link to study page: https://osf.io/q72pz/?view_only=f88f040dec0244f28ba5787f2271693e). As in Study 1, Study 2 consisted of a three-cell experimental design in which participants took the perspective of a negatively stereotyped target while focusing on his difficulties or his resilience, or completed a control perspective-taking task. Consistent with the findings of Study 1, we predicted that focusing on the target’s difficulties would lead Whites to view the Black target as more helpless and less competent. In contrast, we predicted that participants who focused on the target’s resilience would show no increase in their perceptions of the target’s helplessness and instead perceive him as more competent.

### Method

#### Participants

One hundred seventy-five White MTurk workers (62.3% female, 37.7% male; *M*_age_ = 39.63 years, *SD* = 13.94)^5^ participated in a study on the “Development of Reading Materials” in exchange for a cash payment.^6^ As in Study 1, we conducted sensitivity analyses for the focal contrasts using G*Power (Faul et al., 2007). This analysis revealed that Study 2 had 80% power to detect an effect size of *d* = 0.53 for the difficulties vs. control contrast, *d* = 0.52 for the difficulties vs. resilience contrast, *d* = 0.52 for the resilience vs. control contrast.

#### Procedure

The cover story, materials, and procedure were identical to those of Study 1 except for one important change: whereas Study 1 included an article task before the perspective-taking writing activity, Study 2 included only the perspective-taking exercise. Thus, Study 2 included three conditions: a difficulties condition, a resilience condition, and a control condition.

#### Dependent Measures

As in Study 1, participants completed an impression formation task that assessed their perceptions of the target related to helplessness (α = .82) and competence (α = .84).^7^

### Results

#### Analysis Plan

Our first pre-registered prediction was that participants in the difficulties condition would view the target as more helpless relative to both the control condition and the resilience condition. Our second pre-registered prediction was that participants in the difficulties condition would perceive the target as less competent (vs. control), whereas participants in the resilience condition would perceive the target as more competent (vs. control). We tested these predictions using planned comparisons with two-tailed tests.

#### Helplessness

As predicted, we found that focus condition significantly affected ratings of helplessness, *F*(2, 172) = 14.09, *p* < .001. Participants in the difficulties condition viewed the target as more helpless (*M* = 2.61, *SD* = 0.80) than did those in both the control condition (*M* = 1.94, *SD* = 0.70; *t*[172] = 4.96, *p* < .001, *d* = 0.89), and the resilience condition (*M* = 2.05, *SD* = 0.68; *t*[172] = 4.15, *p* < .001, *d* = 0.75). Those in the resilience condition did not significantly differ from those in the control condition, *t*[172] = 0.86, *p* = .39, *d* = 0.16.

#### Competence

Focus condition also influenced participants’ ratings of the target’s competence, *F*(2, 172) = 9.54, *p* < .001. Participants in the difficulties condition (*M* = 3.66, *SD* = 0.78) rated the target as *less* competent than did those in the control condition (*M* = 3.92, *SD* = 0.59; *t*[172] = 2.13, *p* = .034, *d* = 0.38) and the resilience condition (*M* = 4.18, *SD* = 0.55; *t*[172] = 4.37, *p* < .001, *d* = 0.77). Participants in the resilience condition rated the target as *more* competent than did those in the control condition, *t*(172) = 2.23, *p* = .027, *d* = 0.46.

### Discussion

These findings provide additional evidence that the two ways of approaching stigma differentially affect Whites’ perceptions of stigmatized groups. Participants who focused on the Black target’s difficulties viewed him as more helpless and less competent than controls. In contrast, participants who focused on the target’s resilience in the face of his difficulties perceived the target as more competent than controls. Thus, this study replicates the findings of Study 1 and suggests that learning about stereotype threat prior to the perspective-taking exercise is not necessary for the effects of focus condition to emerge.

## Study 3

Studies 1 and 2 suggest that the two ways of approaching stigma have different consequences for peoples’ perceptions of stigmatized targets. A limitation of these studies, however, is that there were subtle differences in scenarios across the experimental conditions. For instance, one scenario in the difficulties condition was “Takes a difficult midterm test while under extra pressure to prove that the negative stereotype about Black people’s abilities isn’t true,” whereas the analogous scenario in the resilience condition was “Takes a difficult midterm test, and what strategies he has learned to use to reduce any anxiety he feels when taking this test.” Thus, although we tried to hold extraneous factors constant, it is possible that our manipulations were confounded with other differences in the content of the scenarios. Moreover, given that the language used in the manipulation instructions (e.g., in which participants were asked to imagine “the kind of help he might need in such situations”) was similar to some of the items used in the measures of helplessness and competence (e.g., “needs help”), one might argue that the findings of Studies 1 and 2 were driven by demand effects. Study 3 addresses these issues by holding the scenarios constant across conditions and manipulating participants’ *construals* of the scenarios, such that participants would construe the target’s experiences in terms of either the difficulties he faces or his resilience in the face of his difficulties. Our predictions, stopping rule, and analysis plan were pre-registered on the Open Science Framework (Open Science Framework, n.d.; link to study: https://osf.io/q72pz/?view_only=0871d485aede4423aba52ee60d98222b).

### Method

#### Participants

Three hundred eighty-four White American MTurk workers (49.5 % male, 50.5% female; *M*_age_ = 37.98, *SD* = 11.24) were recruited to participate in the study via TurkPrime.^8,9^ A sensitivity analysis revealed that Study 3 was adequately powered to detect an effect size of *d* = 0.29.

#### Procedure

The basic procedures used in Study 3 were identical to those of the previous study, with the exception of the focus manipulation, which was altered to address the alternative explanation noted above. In addition, unlike the previous studies, Study 1 only included the two experimental conditions; it did not include a control condition because we did not think that it would be possible to create a “neutral” construal of a stigmatized individual’s experiences. After participants completed the focus manipulation they were presented with a modified version of the perspective-taking activity used in Studies 1 and 2 featuring the same Black target (the Black college student named Tyrone Williams), but holding the content across the two conditions constant.

#### Focus Manipulation

Participants were randomly assigned to a difficulties-focus condition or a resilience-focus condition. Whereas in the previous studies we manipulated participants’ focus by varying the content of the scenarios themselves, in the present study we included the same scenarios across both conditions but manipulated participants’ *construals* of the scenarios. To do so, we first asked participants to complete a series of reading and writing activities that varied in terms of whether they focused on the difficulties that negatively stereotyped individuals face or on their resilience in the face of their difficulties (see online supplemental material for a depiction of each of the components of the focus manipulation).

In both conditions, participants first read an adapted version of the article about stereotype threat that was used in Study 1. Both conditions included a basic overview of stereotype threat, a thought experiment in which participants imagined experiencing stereotype threat, and stories of two individuals’ (a Black college student named Jason and a female engineer named Maria) experiences with stereotype threat. We included examples of female engineers (who also contend with negative stereotypes and stigmatization) because our goal was to manipulate participants’ construals of experiences of stigma in a broad sense, beyond just a single Black male’s experiences of stigma.

In the difficulties condition the content focused on the difficulties associated with experiencing stereotype threat, whereas in the resilience condition the content focused on the target’s resilience in the face of stereotype threat. For instance, in the difficulties condition participants were told that, because of stereotype threat, Maria (the female engineer) “has to deal with this extra pressure to perform well and to not conform to this stereotype” and that “all of this makes the engineering program a very difficult and stressful situation for Maria.” The resilience condition included this content as well but then went on to say that Maria “uses her strengths and skills to manage the extra stress and pressure.”

Both conditions then asked participants to complete a perspective-taking exercise similar to the one used in Study 1, but with a female engineer named “Katherine Parker” as the target of the exercise. The goal of this perspective-taking activity was not to manipulate people’s perceptions of Katherine Parker. Rather, the goal was to further focus participants on the difficulties vs. resilience that stigmatized targets experience to lead participants to construe a second set of scenarios featuring Tyrone Williams in terms of either difficulties or resilience. Moreover, we wanted to include a perspective-taking activity in which the target was still a member of a negatively stereotyped group, but not the same negatively stereotyped group featured in the target scenarios that participants would be asked to complete, to avoid redundancy. Participants were asked to take Katherine’s perspective and write about how she might feel in five different scenarios. In the difficulties condition, the exercise focused on the difficulties Katherine faces as a result of stereotype threat, whereas in the resilience condition, the exercise focused on her resilience in the face of stereotype threat. For instance, one scenario in the difficulties condition was “Meets with her study group to prepare for an exam, and how she feels extra pressure to prove to the group that negative stereotypes about women’s abilities in engineering aren’t true.” The analogous scenario in the resilience condition was “Meets with her study group to prepare for an exam, and how she has learned to perform her best despite the extra pressure to prove to the group that negative stereotypes about women’s abilities in engineering aren’t true.” The goal of these activities was to manipulate participants’ construals of people’s experiences of stereotype threat such that they would evaluate the target scenarios (described below) in terms of the target’s difficulties or their resilience, even though the content of the scenarios was identical across the two conditions.

#### Target Scenarios

After completing the focus manipulation described above, participants responded to the target perspective-taking exercise, featuring the same Black student used in Study 1. Similar to the previous studies, participants were asked to take the perspective of Tyrone Williams and imagine how he might feel in five different scenarios.

Importantly, unlike in the previous studies, the content of the target exercise *did not vary by condition*. The scenarios were designed to be ambiguous enough that they could be interpreted either as a sign of the difficulties the target faces or as a sign of their resilience. For instance, one scenario was “Meets with a few other students to work on a group project for his biology class, and wonders what these students think about his abilities.” We expected that participants assigned to the difficulties-focus condition would construe the scenarios in terms of the difficulties Tyrone faces, whereas those assigned to the resilience condition would construe the scenarios in terms of Tyrone’s resilience. Thus, in this way, we manipulated focus on difficulties vs. resilience, while holding all of the information that participants received about the target constant.

#### Dependent Measures

As in Study 1, the dependent measures consisted of participants’ ratings of Tyrone Williams in terms of helplessness (α = .71) and competence (α = .87).^10^

### Results

#### Helplessness

Consistent with our predictions, participants in the difficulties condition (*M* = 2.27, *SD* = 0.67) viewed the target as more helpless than did those in the resilience condition (*M* = 2.12, *SD* = 0.57), *t*(382) = 2.46, *p* = .015, *d* = 0.24.

#### Competence

In addition, participants in the difficulties condition viewed the target as less competent (*M* = 3.77, *SD* = 0.67) than did those in the resilience condition (*M* = 4.00, *SD* = 0.67), *t*(382) = 3.38, *p* = .001, *d* = 0.34.

### Discussion

Study 3 provides additional evidence to support our model and rules out the possibility that the findings reported in Studies 1 and 2 were due to differences in the ways in which the targets were described beyond focus on difficulties vs. resilience. Indeed, in the present study, we gave participants in both conditions the same information about the target but manipulated their *construals* of his experiences to focus on either his difficulties or resilience. Thus, this study suggests that the different consequences of focusing on difficulties vs. resilience are in fact a result of differences in the types of experiences on which participants are focusing, rather than other differences in content across the two conditions.

Taken together, Studies 1–3 suggest that empathy interventions that focus only on a stigmatized target’s difficulties can backfire by leading non-stigmatized individuals to form paternalistic perceptions of the target. But are the effects of focusing on difficulties limited to empathy interventions? That is, would focusing on a stigmatized target’s difficulties or resilience without also empathizing with the target yield similar effects? Study 4 addressed this question.

## Study 4

Study 4 had two main goals. The first was to replicate the findings of Studies 1–3. The second was to examine whether the effects of focusing on the target’s difficulties vs. resilience would hold regardless of whether participants were asked to empathize with the target. Although our model predicts that the effects of the two approaches should emerge even if participants did not empathize with the target, it is possible that empathy could heighten the effects. Specifically, because empathy interventions ask participants to take the target’s perspective and to try to imagine what the target is feeling, they may increase people’s perceptions of the intensity of the target’s difficulties, thereby increasing the impact of focusing on those difficulties on perceptions of the target. Alternatively, given previous research suggesting that empathy interventions can have primarily positive effects on people’s attitudes towards stigmatized groups (Batson et al., 1997), it is possible that empathizing with the target would *attenuate* the effects of focusing on difficulties vs. resilience. This would suggest that the difference between the difficulties vs. resilience focus would be even larger if participants did not empathize with the target.

To address these questions, we first asked participants to either empathize with a Black target or to take an objective perspective toward the target while focusing on the target’s difficulties, their resilience in the face of their difficulties, or neutral experiences, in a 2 (empathy vs. no empathy) × 3 (difficulties vs. resilience vs. control) between-subjects design. We then examined the extent to which they viewed the target as helpless and competent.

### Method

#### Participants

Eight hundred seventy-five White American Mechanical Turk workers (61.6% female, 38.4% male; *M*_age_ = 37.44 years, *SD* = 11.90) participated in the study in exchange for payment.^11,12^ We conducted a series of power sensitivity analyses using G*Power (Faul et al., 2007) for the interaction and the focal contrasts. These analyses revealed that we had 80% power to detect an effect size of *f* = 0.11 for the interaction and effect sizes of *d* = 0.23 for each of the contrasts reported below.

#### Procedure

Study 4 consisted of a 3 (focus condition: difficulties vs. resilience vs. control) × 2 (empathy condition: empathy vs. no empathy) between-subjects design. The cover story, procedure, materials, and measures were identical to Study 2, except for the addition of an empathy manipulation.

#### Empathy Manipulation

To manipulate empathy for the target, we followed procedures used in previous research (Batson et al., 1997; Galinsky & Moskowitz, 2000). Specifically, the no-empathy condition instructed participants to “try to take an objective perspective toward what is described. Try not to get caught up in how Tyrone feels; just remain objective and detached.” The empathy condition instructed participants to “Imagine a day in the life of this individual as if you were that person, looking at the world through his eyes and walking through the world in his shoes” and to “try to feel the full impact of Tyrone’s experiences and how he feels as a result.”

#### Measures

##### Manipulation Check

To assess the effectiveness of the empathy manipulation, we included two manipulation check items (i.e., “How much do you think you were able to *empathize* with Tyrone? [i.e., to understand and share his feelings]” and “How much do you think you were able to remain *objective and detached* from Tyrone’s feelings?”). These items were rated on a five-point scale (1 = not at all; 5 = an extreme amount). We formed a composite by reversing the second item and averaging across the two items, with higher scores indicating more empathy.

##### Dependent Measures

We assessed perceptions of helplessness (α = .78), and competence (α = .86) with same measures used in Studies 1–3.^13^

### Results

#### Manipulation Check

As expected for the manipulation check, participants in the empathy condition (*M* = 3.40, *SD* = 0.68) reported more empathy for Tyrone than participants in the no-empathy condition (*M* = 2.86, *SD* = 0.69), *F*(1, 869) = 133.80, *p* < .001.^14^

#### Helplessness

There was a main effect of focus condition on helplessness, *F*(2, 869) = 60.83, *p* < .001, and no main effect of empathy condition, *F*(2, 869) = 3.31, *p* = .129. There was also a marginally significant interaction between focus condition and empathy condition, *F*(2, 869) = 2.80, *p* = .061. Given that the interaction was marginally significant, we first report effects for the main effect of focus condition, and then for thoroughness, we report the simple comparisons for the marginally significant interaction.

Tukey’s post hoc comparisons for the main effect of focus condition revealed that participants in the difficulties condition viewed the target as more helpless (*M* = 2.45, *SD* = 0.60) than participants in the control condition (*M* = 1.96, *SD* = 0.56; *p* < .001, *d* = 0.84), and participants in the resilience condition (*M* = 2.07, *SD* = 0.54; *p* < .001, *d* = 0.67). Participants in the resilience condition also saw the target as marginally more helpless than did those in the control condition, *p* = .056, *d* = 0.20.

Breaking down the marginally significant interaction revealed that the effect of focus condition was significant in both the no-empathy condition, *F*(2, 869) = 43.83, *p* < .001, and the empathy condition, *F*(2, 869) = 19.25, *p* < .001. Within each empathy condition, the predicted pattern emerged. In the no-empathy condition, participants in the difficulties condition viewed the target as more helpless (*M* = 2.49, *SD* = 0.60) than controls, (*M* = 1.90, *SD* = 0.55; *p* < .001, *d* = 1.03), and participants in the resilience condition (*M* = 2.01, *SD* = 0.52; *p* < .001, *d* = 0.85). The control and resilience conditions did not differ, *p* = .184, *d* = 0.21. Similarly, in the empathy condition, participants in the difficulties condition viewed the target as more helpless (*M* = 2.42, *SD* = 0.59) than controls, (*M* = 2.03, *SD* = 0.57; *p* < .001, *d* = 0.67), and participants in the resilience condition (*M* = 2.13, *SD* = 0.57; *p* < .001, *d* = 0.50). The control and resilience conditions did not differ, *p* = .323, *d* = 0.17. This pattern suggests that the trend towards an empathy by focus condition interaction was driven by the difference between the focus conditions being slightly stronger in the no-empathy condition.

#### Competence

There was a main effect of focus condition, *F*(2, 869) = 25.87, *p* < .001, no main effect of empathy, *F*(1, 869) = 1.09, *p* = .30 and no empathy by focus condition interaction interaction, *F*(2, 869) = 0.14, *p* = .87.

Participants in the difficulties condition (*M* = 3.56, *SD* = 0.66) perceived the target as less competent than participants in the control condition (*M* = 3.72, *SD* = 0.60; *p* = .005, *d* = 0.25), and the resilience condition (*M* = 3.92, *SD* = 0.57; *p* < .001, *d* = 0.58). In contrast, participants in the resilience condition perceived the target as more competent than did participants in the control condition, *p* < .001, *d* = 0.34.

### Discussion

Study 4 sought to replicate the effects of focusing on a target’s difficulties versus their resilience, as well as to examine whether an empathy manipulation – taking the target’s perspective, or not – is necessary for the effects to emerge. Replicating Studies 1–3, participants who focused on the difficulties that a target faced viewed him as more helpless and less competent than controls, and participants who focused on the target’s resilience viewed him as more competent, regardless of whether they were directed to empathize with him. Unexpectedly, a marginally significant effect emerged in which participants in the resilience condition viewed the target as slightly more helpless than participants in the control condition, which is inconsistent with the results of Studies 1–3.

Breaking down a marginally significant interaction on helplessness to examine the pattern showed that both when participants were directed not to empathize with the target and when they were directed to empathize, a difficulties focus still led them to perceive the target as more helpless than controls.

## Study 5

A limitation of Studies 1–4 is that each included an article about stereotype threat and/or an empathy manipulation or intervention. It remains unclear whether these components are necessary for the effects of focusing on difficulties vs. resilience in the face of stigma. Study 5 addresses these issues by simply asking participants to read about a stigmatized target while focusing on his difficulties, his resilience, or on neutral experiences. In addition, Study 5 examines the effects of focusing on difficulties vs. resilience across a broader set of dependent measures, including feelings of admiration for the target and interest in affiliating with the target in situations in which competence is relevant.

### Method

#### Participants

Six hundred and five White American Mechanical Turk Workers were recruited to participate in the study via TurkPrime (Litman et al., 2017). We excluded participants who did not identify themselves as White, as well as those who completed the survey multiple times. Because there were issues with data quality on TurkPrime at the time that we ran this study, we also included four Winograd Schema Challenge questions (Bender, 2015) to screen out poor-quality data (e.g., “data farmers”). Participants were asked to complete two randomly selected questions out of the four total questions; we excluded those who answered one or more of the questions incorrectly. Our final sample consisted of 488 White participants (*M*_age_ = 33.75 years, *SD* = 10.57; 46.7% men, 53.1% women, 0.2% another gender).

#### Procedures

The procedures of this study were similar to those of the previous studies. Participants were first asked to complete a modified version of the focus manipulation, in which they were randomly assigned to a difficulties condition, a resilience condition, or a control condition. They were then asked to complete the dependent measures.

#### Focus Manipulation

As in Studies 1–4, participants were asked to read about the experiences of Tyrone Williams, a Black college student. Unlike the previous studies, however, the manipulation did not include a stereotype threat article or a perspective-taking exercise. Rather, they were simply asked to “Read about the life about Tyrone Williams.” They were then presented with the same five scenarios used in Studies 1, 3 and 4 describing Tyrone’s experiences. In the difficulties condition, the scenarios described difficulties Tyrone faces as a result of stigma. In the resilience condition, the scenarios described his resilience in the face of difficulties. In the control condition, the scenarios described neutral experiences. In contrast to the previous studies, participants were not asked to write a response to the scenarios; they were simply asked to read them.

#### Dependent Measures

##### Helplessness and Competence

Perceptions of the target in terms of helplessness (α = .76) and competence (α = .87) were assessed with the same measures used in Studies 1–4.

##### Admiration

Our measure of admiration was adapted from previous research (Cuddy et al., 2007). Participants were asked to respond to five items assessing their feelings toward the target. Specifically, they were asked to report the extent to which each of the following items described their feelings towards Tyrone: Admiring, respectful, proud, fond, inspired (1 = not at all, 5 = an extreme amount). We formed a composite by averaging across the five items (α = .91).

##### Affiliation

To assess interest in affiliating with the target in domains in which competence is relevant, we asked participants how willing they would be to engage in different activities with Tyrone in work and academic settings. Specifically, participants responded to the following five items: “How interested would you be in working on an intensive, three-month project with Tyrone?”; “How interested would you be in joining a work group with Tyrone?”; “How interested would you be in asking Tyrone to provide feedback on a report you wrote?”; “How interested would you be in having Tyrone as a partner on a work project?”; “How likely would you be to ask Tyrone for help on a task or assignment if you were struggling?” Responses were given on a five-point scale (1 = not at all interested, 5 = extremely interested). A composite was formed by averaging across the five items (α = .95).

### Results

#### Analysis Plan

Consistent with the previous studies, for each of the dependent measures, we conducted a one-way ANOVA and then a series of planned contrasts to examine the effects of the two focus conditions on each of the dependent measures. We predicted that participants in the difficulties-focus condition would view the target as more helpless and less competent and also show less admiration and less interest in affiliating with the target, relative to those in the control condition and those in the resilience condition. In contrast, we predicted that participants in the resilience-focus condition would view the target as more competent and report more admiration and more interest in affiliating with the target, relative to those in the control condition.

#### Helplessness

Consistent with our predictions, focus condition affected ratings of helplessness, *F*(1, 485) = 94.92, *p* < .001. Participants in the difficulties-focus condition (*M* = 2.48, *SD* = 0.65) perceived the target as more helpless than did those in the resilience condition (*M* = 1.89, *SD* = 0.52; *t*[485] = 9.74, *p* < .001, *d* = 1.00) and the control condition (*M* = 1.68, *SD* = 0.43; *t*[485] = 13.37, *p* < .001, *d* = 1.45). Participants in the resilience condition also rated the target as more helpless than did those in the control condition (*t*[485] = 3.62, *p* < .001, *d* = 0.45), which is consistent with the results of Study 4.

#### Competence

Focus condition also affected ratings of the target’s competence, *F*(1, 485) = 21.47, *p* < .001. Participants in the difficulties condition (*M* = 3.62, *SD* = 0.71) rated the target as less competent than did those in the resilience condition (*M* = 4.07, *SD* = 0.61; *t*[485] = −6.55, *p* < .001, *d* = 0.69), and those in the control condition (*M* = 3.84, *SD* = 0.54; *t*[485] = −3.30, *p* = .001, *d* = 0.36). In contrast, participants in the resilience condition rated the target as more competent than did those in the control condition, *t*(485) = 3.23, *p* = .001, *d* = 0.395.

#### Admiration

In addition, focus condition affected feelings of admiration for the target, *F*(1, 485) = 5.25, *p* = .006. Participants in the difficulties condition (*M* = 3.39, *SD* = 0.89) felt less admiration for the target relative to those in the resilience condition (*M* = 3.70, *SD* = 0.72; *t*[485] = −3.16, *p* = .002, *d* = 0.35). Participants in the difficulties condition did not significantly differ from those in the control condition (*M* = 3.48, *SD* = 0.80; *t*[485] = −1.03, *p* = .30, *d* = 0.11), however. In contrast, participants in the resilience condition reported greater feelings of admiration for the target than did those in the control condition, *t*[485] = 2.18, *p* = .03, *d* = 0.26.

#### Affiliation

Finally, focus condition affected interest in affiliating with the target in competence-relevant domains, *F*(1, 485) = 6.34, *p* = .002. Participants in the difficulties condition (*M* = 3.45, *SD* = 1.04) were less interested in affiliating with the target than were participants in the resilience condition (*M* = 3.78, *SD* = 0.86; *t*[485] = −3.20, *p* = .001, *d* = 0.34). As above, participants in the difficulties condition did not significantly differ from those in the control condition (*M* = 3.48, *SD* = 0.81; *t*[485] = −0.32, *p* = .75, *d* = 0.03), however. Participants in the resilience condition showed greater interest in affiliating with the target than did those in the control condition, *t*(485) = 2.94, *p* = .003, *d* = 0.35.

### Discussion

Study 5 provides additional evidence that focusing on a stigmatized target’s difficulties vs. resilience can have different consequences for perceptions of the target. Indeed, consistent with Studies 1–4, we found that participants who focused on the target’s difficulties perceived him as more helpless and less competent, whereas those who focused on the target’s resilience perceived him as more competent. Moreover, the focus manipulation used in Study 5 did not include a stereotype threat article or an empathy task, suggesting that these components are not necessary for the effects of focusing on difficulties vs. resilience to emerge.

In addition, Study 5 suggests that the two ways of approaching stigmatization may affect not only perceptions of helplessness and competence, but also feelings of admiration for the target and interest in affiliating with the target in domains in which competence is relevant. Specifically, participants who focused on the target’s difficulties reported less admiration for the target and less interest in affiliating with the target relative to those who focused on the target’s resilience. In contrast, participants who focused on the target’s resilience showed relatively more admiration for the target and more interest in affiliating with him.

Participants in the difficulties condition did not report less admiration and interest in affiliation relative to those in the control condition. It is possible that this pattern emerged because the control condition was less descriptive and contained less information about the target relative to the two experimental conditions. As such, although participants in the control condition may not have seen reason to doubt the target’s competence, they may also not have had strong reasons to admire the target or to want to affiliate with him in competence-relevant domains. Surprisingly, participants in the resilience-focus condition viewed the target as more helpless than participants in the control condition, a finding that did not emerge in the other studies except with marginal significance in Study 4. One possible explanation is that, without the stereotype threat information from Studies 1–4, peoples’ stereotypical perceptions about the helplessness of stigmatized individuals emerge from even a resilience-focused discussion of stigma.

Nevertheless, Study 5 provides additional evidence of the different consequences of focusing on difficulties vs. resilience, and suggests that these effects do not emerge only in the context of reading about stereotype threat or completing an empathy task.

## General Discussion

Conventional wisdom suggests that increasing non-stigmatized or majority group individuals’ understanding of the experiences of members of stigmatized groups will lead to increased positive perceptions of stigmatized individuals and, therefore, more positive intergroup relations. Yet the present research suggests that when such efforts focus narrowly on stigmatized individuals’ experiences of struggle and hardship, they may backfire by leading to more paternalistic perceptions of targets. Indeed, the five studies reported here suggest that focusing only on the difficulties that stigmatized individuals face may lead non-stigmatized individuals to take a paternalistic view of stigmatized targets. The present research also suggests that focusing more broadly on targets’ resilience in the face of their difficulties can improve non-stigmatized individuals’ perceptions of stigmatized targets.

In Studies 1 and 2, we examined the consequences of an empathy intervention in which White participants were asked to take the perspective of a Black student while focusing on his difficulties or on his resilience in the face of his difficulties (vs. a control condition). Whites who focused on the student’s difficulties subsequently viewed him as more helpless and less competent relative to those in the control condition and the resilience condition. In contrast, Whites who focused on the student’s resilience in the face of his difficulties perceived him as more competent than those in the control condition. In Study 3, we ruled out differences in the ways in which the target was described across conditions as an alternative explanation for the findings. To do so, we held constant the information participants received about the target and manipulated their *construals* of that information. Once again, participants in the difficulties condition perceived the target as more helpless and less competent than did those in the resilience condition. In Study 4, we replicated and extended these findings by examining whether it was necessary to instill empathy for the effects to emerge. In this study, we found that, regardless of whether they were asked to empathize with the target or take an objective perspective, Whites who focused on the Black target’s difficulties viewed him as more helpless and less competent than those in a control condition, whereas those who focused on the target’s resilience viewed him as more competent than controls. Thus, although we used interventions to create empathy for targets of stereotype threat as the primary operationalization of the two ways of approaching stigma, Study 4 suggests that the consequences of these two approaches extend beyond the context of empathy interventions. Finally, in Study 5, we examined whether simply reading about a stigmatized target’s difficulties or resilience, without also reading about stereotype threat, would have similar effects to the focus manipulations used in Studies 1–4. Additionally, we measured new outcomes – ratings of affiliation and admiration. This study replicated the findings of Studies 1–4 and found that participants who focused on difficulties still saw the target as more helpless and less competent than controls. Participants who focused on resilience saw the target as more competent and reported more admiration and interest in affiliation relative to controls. Taken together, these four studies suggest that the two ways of approaching stigma – the difficulties-focused approach vs. the resilience-focused approach – have different consequences for people’s perceptions of stigmatized individuals; whereas the resilience-focused approach leads to respectful views of stigmatized targets, the difficulties-focused approach leads to paternalistic views of targets.

### Limitations and Future Directions

Although the present research provides consistent evidence regarding the consequences of the two approaches to stigma, there are limitations and caveats worth noting. One such limitation is that our dependent measures were limited to people’s self-reported perceptions of racial minority targets. Do the two approaches to stigma differentially affect people’s behaviors towards racial minority targets as well? To the extent that paternalistic perceptions engender more paternalistic and less respectful behaviors towards targets, our model of stigma could have important implications for interracial interactions and for outcomes experienced by racial minorities. Indeed, previous research has shown that, whereas racial majority group members primarily seek to be *liked* during interracial interactions, racial minorities seek to be *respected* (Bergsieker et al., 2010). Thus, if the difficulties-focused approach to stigma leads Whites to behave in more disrespectful ways toward racial minorities, it could undermine interracial interactions and exacerbate racial disparities by creating self-fulfilling prophecies (Word et al., 1974).

Along similar lines, the present research examines people’s perceptions of stigmatized *individuals*. Thus, one outstanding question is whether people would generalize from their perceptions of stigmatized individuals to the stigmatized *groups* to which these individuals belong, given that some such approaches are likely to focus on groups overall. Although we did not assess this possibility in these studies, doing so will be an important task for future research.^15^ We caution that there may be a number of reasons why this relationship may not be entirely straightforward. Specifically, although participants may be comfortable reporting negative perceptions of a single stigmatized target, they may be less willing to report negative perceptions of stigmatized groups as a whole because of concerns about appearing prejudiced.

A third limitation concerns the similarity of some of the items in the measures of helplessness and competence to the wording of the manipulations used in Studies 1 and 2. Specifically, some of the instructions used in the manipulations in Studies 1 and 2 (e.g., “[imagine] what kind of help he might need in these situations”) were very similar to the items used in the main dependent measures (e.g., “needs help”), which may have created demand effects. We do not think that the findings of Studies 1 and 2 can be explained entirely by demand effects, however. Indeed, we removed any wording that was similar to the dependent measures in Studies 4 and 5 and successfully replicated the findings of Studies 1 and 2. Moreover, in Study 3, we gave participants identical information about the targets across the two conditions and found consistent effects.

Finally, it is important to note that we are not attempting to characterize the two approaches to stigma as wholly positive or negative. Indeed, there may be positive effects of focusing on a stigmatized target’s difficulties that we have not examined in the present research, such as increasing people’s empathic concern for stigmatized individuals or increasing pro-social behaviors towards stigmatized groups (Batson et al., 2002; Batson et al., 1997). Similarly, there may be negative consequences associated with the resilience-focused approach. For instance, learning about a stigmatized individual’s resilience in the face of stigma may lead non-stigmatized individuals to downplay the consequences of stigma or to more readily engage in rationalization of stigma (e.g., by engaging in system justification; Jost & van der Toorn, 2012). Thus, we do not suggest that non-stigmatized individuals should *never* focus on the difficulties faced by members of stigmatized groups. Neither do we suggest that focusing on their resilience is a panacea for improving intergroup relations. Rather, we highlight one underappreciated negative consequence of efforts to educate non-stigmatized individuals about stigmatization and group-based disadvantage and suggest that such efforts should avoid taking a narrow focus on stigma in which members of stigmatized groups are defined by their struggles and hardships. Instead, discussions of stigma should attempt to foster a broader understanding of stigmatized individuals in which their full humanity is recognized and appreciated.

### Implications

The present research has important implications for theories of intergroup perception. To our knowledge, this work is the first to examine how learning about the difficulties faced by members of stigmatized groups affects people’s perceptions of stigmatized individuals. Although one might think that learning about the difficulties stigmatized individuals face would lead to entirely positive perceptions of these individuals, our research suggests that doing so can lead people to form paternalistic impressions of stigmatized individuals. Ultimately, the difficulties-focused approach may lead to worse outcomes for stigmatized groups insofar as it causes non-stigmatized individuals to behave in more disrespectful ways towards stigmatized individuals or to withhold opportunities and resources from them. In this way, the difficulties-focused approach could ultimately reinforce stigmatization over time. In contrast, focusing on these individual’s resilience in the face of their difficulties may offer a way for non-stigmatized individuals to learn about the experiences of stigmatized groups, and even increase their respect for them, without incurring the negative costs of the difficulties-focused approach.

This work also has implications for research on empathy in intergroup contexts. Although a growing body of research has shown that empathy can have negative effects in intergroup contexts (Vorauer et al., 2009; Vorauer & Sasaki, 2009, 2012), both conventional wisdom and the vast majority of empirical research suggest that empathy has positive consequences for intergroup relations (Batson & Ahmad, 2009). Our research suggests that the effects of empathizing with a member of a stigmatized group may depend in part on with which aspects of the target’s experiences are emphasized. If the focus is only on the target’s struggles and difficulties, empathy will likely be harmful. If the focus is on the target’s resilience in the face of their difficulties, empathy could prove beneficial to ratings of competence. Thus, the present research may provide valuable information about when empathy interventions are likely to have entirely positive vs. at least partially negative effects for people’s perceptions of stigmatized individuals.

The current work may also be relevant to the literature on dependency vs. autonomy-oriented helping (see Nadler, 2002 for a review). This literature suggests that providing *dependency-oriented* help to members of lower-status groups can act as a status-maintaining mechanism because it places the help-recipients in a low-status, dependent position relative to the help-provider. In contrast, *autonomy-oriented helping* focuses on empowering individuals to take an active role in addressing their struggles, and is therefore less likely to undermine the recipient’s social status. It is possible that the two approaches to stigmatization examined in the present research may motivate different forms of helping; whereas the difficulties-focused approach may motivate dependency-oriented helping, the resilience-focused approach may motivate autonomy-oriented helping. Future work should explore the connections between these two lines of research.

Finally, this research has important practical implications. Reducing stigmatization and improving people’s perceptions of stigmatized groups has become an increasingly important issue in society, and there has been a push for programs to increase diversity and reduce the negative effects of stigma across many different types of organizations. It would not be surprising if at least some of these programs have incorporated efforts to educate non-stigmatized individuals about the experiences of members of stigmatized groups. For instance, in one well-known example, Starbucks closed all of its stores for a one-day diversity and inclusion training. As part of this training, Starbucks employees watched a short video entitled “The Story of Access,” that focused on primarily on the struggles and difficulties that Black individuals face, with no discussion of Black people’s broader experiences including their resilience in the face of difficulty. Although these types of programs may be well-intentioned, the present work suggests they may ultimately lead people to form more negative perceptions of stigmatized individuals because they focus exclusively on the difficulties that many members of stigmatized groups face. With a relatively subtle shift, however, such efforts could produce their intended effects. Specifically, organizations should be mindful of the ways they describe the experiences of stigmatized individuals and avoid characterizing them as in need of help or lacking resilience. Instead, organizations should explicitly acknowledge the resilience of stigmatized individuals; doing so may create more respectful environments for stigmatized groups and allow them to thrive.

## Conclusion

In a society in which group-based stigmatization is an unfortunate reality, the question of how best to foster understanding across group lines is a critical concern. Conventional wisdom suggests that one way to do so is to educate non-stigmatized individuals about the barriers and disadvantages faced by members of stigmatized groups. Yet our research suggests that such efforts must be made with caution. To the extent that these efforts focus narrowly on stigmatized individuals’ experiences of struggle and hardship, they may hinder rather than enhance understanding, and ultimately widen rather than narrow existing group divides.

## Supplemental Material

sj-pdf-1-gpi-10.1177_13684302211009590 – Supplemental material for When seeing stigma creates paternalism: Learning about disadvantage leads to perceptions of incompetence Click here for additional data file. Supplemental material, sj-pdf-1-gpi-10.1177_13684302211009590 for When seeing stigma creates paternalism: Learning about disadvantage leads to perceptions of incompetence by Stephanie L. Reeves, Crystal Tse, Christine Logel and Steven J. Spencer in Group Processes & Intergroup Relations

## Notes

1. In the interest of brevity, we report several exploratory measures that were included in Study 1 and in Study 4 in the online supplemental materials. All focal dependent measures are reported in the manuscript.

2. Sample size was determined based on power analyses using estimated effect sizes from pilot studies. No data collection occurred after the data were analyzed.

3. It is possible that the inclusion of this language in the two experimental conditions (i.e., “the kind of help he might need in such situations” in the difficulties condition and “what he could teach you about dealing with challenging situations” in the resilience condition) created a demand effect, given its similarity to the language used in the dependent measures. We removed this language in later studies. Given the consistency of the findings across studies, the inclusion of this language does not appear to be driving the results.

4. A number of other exploratory measures were included and are discussed in more detail in the online supplemental material. In addition, one additional item (“uncertain”) was included with the measures of helplessness and competence; this item was included as a filler item and was therefore not analyzed.

5. We collected data from 203 participants. We ultimately ended up with 175 participants: 19 participants completed the survey multiple times and their duplicate entries were excluded, and nine participants were excluded because they appeared to “fake” being White on the prescreening survey (i.e., they completed the prescreening survey several times until they met the eligibility criteria). When these participants are included, all of the primary findings hold, with the exception of the effect of the resilience condition vs. the control condition on perceptions of competence; this contrast remains directional, but non-significant (*p* = .43). Exclusion rates did not vary by condition (*p* = .61).

6. Sample size was determined based on a power analysis using the effect sizes for the effect of the difficulties-focus condition (vs. resilience) from pilot studies. No data collection occurred after data analysis.

7. “Uncertain” and “happy” were included as filler items and were not analyzed.

8. We originally recruited 401 participants, five of these were excluded because they completed the survey multiple times. Twelve participants were excluded because they indicated that their race/ethnicity was not White. Exclusion rates did not vary by condition (*p* = 0.32).

9. Sample size was determined based on a power analysis using the effect sizes for the effect of the difficulties-focus condition (vs. resilience) from pilot studies. No data collection occurred after data analysis.

10. On an exploratory basis, we also assessed participants’ support for various policies to support negatively stereotyped groups. These measures are included and discussed in more detail in the online supplemental materials. In addition, as in the previous studies, “uncertain” was included as a filler item for the helplessness/competence measures.

11. Sample size was determined based on an initial pilot study with the same design that yielded a trending but non-significant main effect of the empathy manipulation (*n* = 451; see online supplemental materials for more information about this study). We thought that our failure to find a significant main effect of empathy may have been due to insufficient statistical power: we therefore decided to run Study 4 with approximately double the *n* of the initial pilot study. No data collection occurred after we analyzed the data.

12. We ultimately recruited 902 participants, however, our final sample consisted of 875 participants; 27 participants were excluded because they had duplicate entries or because they completed the pre-screening test multiple times and had previously indicated a race/ethnicity other than White. All of the primary results hold when these participants are included. Exclusion rates did not vary by condition (*p*s > .05).

13. As in Studies 1 and 2, “uncertain” was included as a filler item and was not analyzed.

14. Although the purpose of the manipulation check was to verify that the empathy manipulation was successful, we did examine the full empathy by focus condition model. We report it in the online supplemental materials.

15. But see the online supplemental materials, Study S1, for an exploratory pilot study that did not find effects of a similar manipulation on perceptions of Black people as a group, and analysis of an exploratory outcome from Study 3, which found no effect for support of relevant policies.
