# Supplementary material for: When seeing stigma creates paternalism: Learning about disadvantage leads to perceptions of incompetence
Source: Group Process Intergroup Relat. 2021 May 29;25(5):1202–22. doi: 10.1177/13684302211009590 (PMC9310139; doi:10.1177/13684302211009590)
Supplement: sj-pdf-1-gpi-10.1177_13684302211009590 – Supplemental material for When seeing stigma creates paternalism: Learning about disadvantage leads to perceptions of incompetence [file sj-pdf-1-gpi-10.1177_13684302211009590.pdf]

SUPPLEMENTARY MATERIAL FOR:

When seeing stigma creates paternalism: Learning about disadvantage leads to perceptions of incompetence

Overview

This online supplement contains the following information:

- Exact manipulations used in Studies 1-5.
- Target scenarios from Study 3.
- Exact items from the dependent measures used in Studies 1-5.
- Rates of attrition by condition for Studies 1-5.
- Information regarding additional exploratory dependent measures used in Studies 1 & 3.
- A supplementary note regarding sample sizes and a supplementary analysis from Study 4
- Methods and results for five supplementary studies not included in the paper because of space constraints.
- Brief discussion of the supplementary studies.

Experimental manipulations used in Studies 1-4

Studies 1-2 & 4

Control focus condition.

Article task (Study 1 only).

Please read the following article summary. You will complete a short writing activity afterwards. The summary was taken from the Plants & Animals section of the ScienceDaily, a web site devoted to the news in science research.

How Some Plants Spread Their Seeds: Ready, Set, Catapult

ScienceDaily (Nov. 4, 2010) — Catapults are often associated with a medieval means of destruction, but for some plants, they are an effective way to launch new life. Dispersing seeds greater distances by catapulting can provide advantages, including the establishment of populations in new environments and escape from certain threats.

In new work published in the recent October issue of *American Journal of Botany*, Dr. Ellerby, students, and postdoctoral researcher Shannon Gerry at Wellesley College measured the mechanics involved in catapulting seeds for the plant species, *Cardamine parviflora*.

"While plants are generally thought of as still organisms, many of them are capable of spectacularly rapid movements," stated Ellerby. For *Cardamine parviflora*, organs within the plant rapidly coil outward catapulting the seeds away from it. The entire coiling and launching process is completed in around 5 milliseconds -- faster than the blink of an eye.

This incredible speed and high energy storage present a challenge for the researchers for videotaping the process. "These seed pod catapults are on a hair trigger," said Ellerby. "Successfully positioning them in front of our high-speed camera without them exploding requires an incredibly steady hand."

Seed launching has evolved in a number of groups. Researchers can understand the evolution of this mechanism by comparing the processes of seed dispersal of fruits and seeds between plants using this launching method and those that do not.

Seed dispersal has been studied extensively in the model plant *Arabidopsis thaliana*, a close relation to *Cardamine parviflora*. *Arabidopsis thaliana* does not disperse its seeds via catapulting. Instead, the seeds are dropped to the ground. Despite these differences in seed dispersal processes, the plant parts of *Cardmamine parviflora* and *Arabidopsis thaliana* are similar. One difference is that there is a second layer of plant material in *Cardamine parviflora* that is absent in *Arabidopsis thaliana*. This additional layer likely plays a role in the launching.

"Ultimately it will be important to analyze the structures at a tissue and cellular level to determine precisely how they store such impressive amounts of energy," Ellerby said. "This could inform the design of human-engineered structures for absorbing or storing elastic energy."

Perspective-taking exercise (Studies 1 -2 &4).

Writing Activity

Now we'd like you to write a brief summary about the life of **Tyrone Williams**, a student in his second year of college.

Imagine a day in the life of this individual as if you were that person, **looking at the world through his eyes** and **walking through the world in his shoes**.

For each of the scenarios below, please write 1 sentence about how Tyrone might feel in these situations: As you do so, try to **feel** the full impact of Tyrone’s experiences and how he **feels** as a result.

- Goes to a political science lecture.
- Meets with his English professor to talk about his term paper.
- Meets with a few other students to work on a group project for his biology class.
- Takes a difficult midterm test.
- Gives a presentation in history class.

Difficulties focus condition.

Article task (Study 1 only).

Please read the following article taken from Claude Steele's book (Steele, 2011), *Whistling Vivaldi: How stereotypes affect us and what we can do.* You will complete a short writing activity afterwards.

Consider the following thought experiment:

Imagine that you arrived late to your family reunion.

Thinking that everyone else has already eaten, you head to the buffet table and load your plate with food.

But as you leave the buffet table, you notice a group of people who haven't eaten a bite yet. They look at the few remains on the food table and then at your plate filled with food. You try to explain, but your effort is lost in the noise of the crowd and the music. You hear them say as they go by, "Those Smiths are so selfish."

Based on this "misunderstanding" you might fear that your immediate family is going to be judged by the stereotype that they are selfish. Every time you do something that could be interpreted in light of that negative stereotype—such as volunteering to dry the dishes instead of wash, deciding how much to tip the pizza man—you are at risk of confirming their stereotype that your family is selfish.

Such suspicion, and the unfair interpretation of your behavior, can make you feel uncomfortable. It could distract you and interfere with your interactions with other people. This experience is related to the experience of a threat from a negative stereotype – researchers call this "Stereotype threat".

But imagine how stereotype threat could get even worse. Suppose that the negative view of you had nothing to do with your own behavior but came from what people thought the group that you belong to, such as your race. Suppose that more people than just your relatives knew about this negative view - say everyone in society knew. Suppose that the negative views reflected not your table manners, but very important abilities that are important to getting ahead, for example, your intelligence in general. And, suppose that the new view applied to you in exactly those situations that were most critical to your success in school and in society – when you take a test, when you do a lab project.

Stereotype threat describes the experience of being at risk of being judged through the lens of a negative stereotype. Everyone has different identities that have stereotypes attached to them – an older person might be judged by the stereotype that she is forgetful, a White man might be judged by the stereotype that white men can't dance, a person with glasses might be judged by the nerd stereotype.
To see this in your own life, think of the important settings in your life: Your school, your workplace, your family. Imagine a situation in which your identity does matter in the situation, that it affects how you see things, whom you identify with, how you react emotionally to events in the setting, and how you perform. Here is an example (a true story of a friend of the writer):

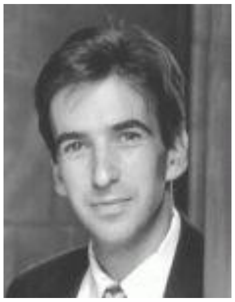
Geoff

A college student named Geoff was one of the only two whites in an African American political science class composed of mostly black and other minority students. He worried about fitting in because he was White, and he was concerned that he could be judged because of it - that if he said anything that revealed an ignorance of African American experience, or a confusion about how to think about it, then he could well be seen as racially insensitive. However, if he said nothing in class, then he could escape the suspicion of his fellow students. His condition made him feel his racial identity, his whiteness, in that time and place something he hadn't thought much about before.

Here is an example relevant to engineering (also a friend of the writer):

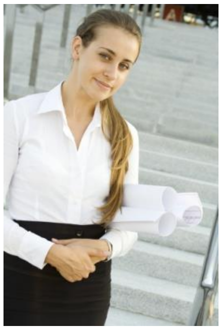
Maria

An engineering student named Maria wanted to do well in her program for the same reasons that everyone does – to get a good job, to meet her personal standards, to make her parents proud. But as a Hispanic woman, she spends every day being outnumbered by men in her engineering classes. Being a Hispanic woman puts her at risk of confirming the stereotype that women and Hispanics are not good at math and other quantitative skills. In addition to all the reasons she wants to do well in her courses, she has to deal with this extra pressure to perform well and to not conform to this stereotype.

Perspective-taking exercise (Studies 1 -2 &4).

Writing Activity

Now we'd like you to write a brief summary about the life of **Tyrone Williams**, a student in his second year of college.

Imagine a day in the life of this individual as if you were that person, **looking at the world through his eyes** and **walking through the world in his shoes**.

For each of the scenarios below, please write 1 sentence about how Tyrone might feel in these situations: As you do so, try to **feel** the full impact of Tyrone's experiences and how he **feels** as a result.[1]

Try to imagine how much more **difficult** his experiences are than the typical student, and how **stressful** or **challenging** it might be for him.

- Meets with his English professor to talk about his term paper, and how he might worry that the professor might judge his abilities in light of negative stereotypes about Black people.
- Meets with a few other students to work on a group project for his biology class, and how he might worry that his classmates are judging his abilities based on negative stereotypes about Black people.
- Takes a difficult midterm test while under extra pressure to prove that the negative stereotype about Black people's abilities isn't true.
- Gives a presentation in history class, and how stressful or challenging the situation can be for him.

Resilience focus condition.

Article task (Study 1 only). *Article task from the resilience focus condition was identical to the one used in the difficulties focus condition and is therefore not included here.*

Perspective-taking exercise (Studies 1 -2 &4).

Writing Activity

Now we'd like you to write a brief summary about the life of **Tyrone Williams**, a student in his second year of college.

Imagine a day in the life of this individual as if you were that person, **looking at the world through his eyes** and **walking through the world in his shoes**.

For each of the scenarios below, please write 1 sentence about how Tyrone might feel in these situations: As you do so try to **feel** the full impact of Tyrone's experiences and how

[1] The instructions for the difficulties-focused condition and the resilience-focused condition used in Studies 1-2 differed slightly from Study 3. Specifically, they included the following additional instructions, respectively: "Try to imagine how much more difficult his experiences are than the typical student, and the kind of help he might need in such situations"; "Try to imagine how much stronger he will become from these difficult experiences, and what he could teach you about dealing with challenging situations." Because of their conceptual proximity to the dependent measures, these instructions were not included in the manipulations used in Study 3 in the paper. Given the consistency of the findings across all three studies, the inclusion of these instructions in Study 1-2 does not appear to be driving the results.

he **feels** as a result.

Try to imagine how he might **overcome the challenges he faces**, and how much **stronger** he will become from these difficult experiences.

- Goes to a political science lecture, and he is the only Black student in the class.

- Meets with his English professor to talk about his term paper, and what strategies he uses to get the most out of the feedback despite the professor's potential doubts about his ability.

- Meets with a few other students to work on a group project for his biology class, and what he could teach them about working well together even when they have concerns about everyone working hard on the project.

- Gives a presentation in history class, and what he could teach other students about concerns about how others will perceive them in front of the class.

- Writes a difficult midterm test, and what strategies he has learned to use to reduce any anxiety he feels when taking this test.

No-empathy condition instructions. *(Study 4 only; the scenarios for the no-empathy conditions are identical to their analogous empathy conditions and thus are not included below).*

Control + no-empathy condition.

Writing Activity

Now we'd like you to write a brief summary about the life of **Tyrone Williams**, a student in his second year of college.

Imagine a day in this individual's life.

For each of the scenarios below, please write 1 sentence about Tyrone in these situations: As you do so, try to **t**ake an **objective perspective** toward what is described. Try **not** to get caught up in how Tyrone feels; just remain **objective** and **detached.**

Difficulties focus + no-empathy condition.

Writing Activity

Now we'd like you to write a brief summary about the life of **Tyrone Williams**, a student in his second year of college.

Imagine a day in this individual's life.

For each of the scenarios below, please write 1 sentence about Tyrone in these situations: As you do so, try to **t**ake an **objective perspective** toward what is described. Try **not** to get caught up in how Tyrone feels; just remain **objective** and **detached.**

Try to imagine how much more **difficult** his experiences are than the typical student, and how **stressful** or **challenging** it might be for him. Again, please try to remain **objective** and **detached** as you do so.

Resilience focus + no-empathy condition.

Writing Activity

Now we'd like you to write a brief summary about the life of **Tyrone Williams**, a student in his second year of college.

Imagine a day in this individual's life.

For each of the scenarios below, please write 1 sentence about Tyrone in these situations: As you do so, try to take an **objective perspective** toward what is described. Try **not** to get caught up in how Tyrone feels; just remain **objective** and **detached**.

Try to imagine how he might **overcome the challenges he faces**, and how much **stronger** he will become from these difficult experiences. Again, please try to remain **objective** and **detached** as you do so.

Study 3

Difficulties focus condition.

Article

Please read the following article, taken from Claude Steele's book, *Whistling Vivaldi: And other clues to how stereotypes affect us*. You will complete a short writing activity afterwards.

Consider the following thought experiment:

Imagine that you arrived late to your family reunion.

Thinking that everyone else has already eaten, you head to the buffet table and load your plate with food. But as you leave the buffet table, you notice a group of people who haven't eaten a bite yet. They look at the few remains on the food table and then at your plate filled with food. You try to explain, but your effort is lost in the noise of the crowd and the music. You hear them say as they go by, "Those Smiths are so selfish."

Based on this "misunderstanding" you might fear that your immediate family is going to be judged by the stereotype that they are selfish. Every time you do something that could be interpreted in light of that negative stereotype - such as volunteering to dry the dishes instead of wash, deciding how much to tip the pizza man - you are at risk of confirming their stereotype that your family is selfish. Such suspicion, and the unfair interpretation of your behavior, can make you feel uncomfortable. It could distract you and interfere with your interactions with other people. This experience is related to the experience of a threat from a negative stereotype - researchers call this "Stereotype threat."

But imagine how stereotype threat could get even worse. Suppose that the negative view of you had nothing to do with your own behavior but came from what people thought the group that you belong to, such as your race. Suppose that more people than just your relatives knew about this negative view - say everyone in society knew. Suppose that the negative views reflected not your table manners, but very important abilities that are important to getting ahead, for example, your intelligence in general. And, suppose that the new view applied to you in exactly those situations that were most critical to your success in school and in society - when you take a test, when you do a lab project.

Stereotype threat describes the experience of being at risk of being judged through the lens of a negative stereotype. Everyone has different identities that have stereotypes attached to them - an older person might be judged by the stereotype that she is forgetful, a White man might be judged by the stereotype that white men can't dance, a person with glasses might be judged by the nerd stereotype.

To see this in your own life, think of the important settings in your life: Your school, your workplace, your family. Imagine a situation in which your identity does matter in the situation, that it affects how you see things, whom you identity with, how you react emotionally to events in the setting, and how you perform. **Think about how difficult it would be to experience the extra stress and pressure that the negative stereotype creates.**

Here is an example (a true story of a friend of the writer):

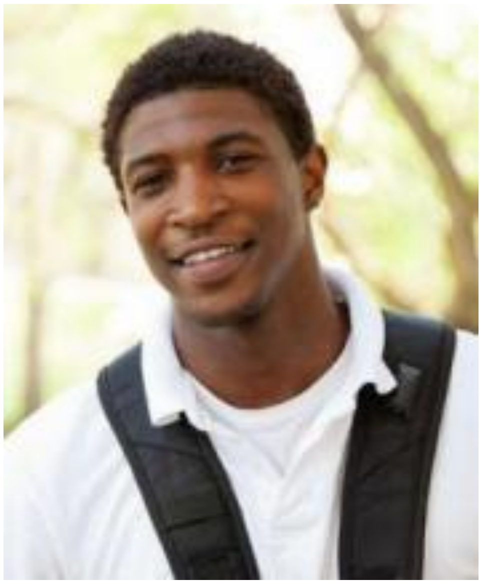

Jason

A college student named Jason was one of the only two Black students in an advanced writing class. In this context he worried about fitting in because he was Black, and he was concerned that he could be judged because of it - that if he said anything wrong or made any mistakes, he could confirm negative stereotypes about Black people. However, if he said nothing in class, then he could escape the suspicion of his fellow students. Because of these concerns, Jason experiences **extra stress** and **anxiety** in the class. **His experiences are much more difficult than the average student.**

Here is an example relevant to engineering (also a friend of the writer):

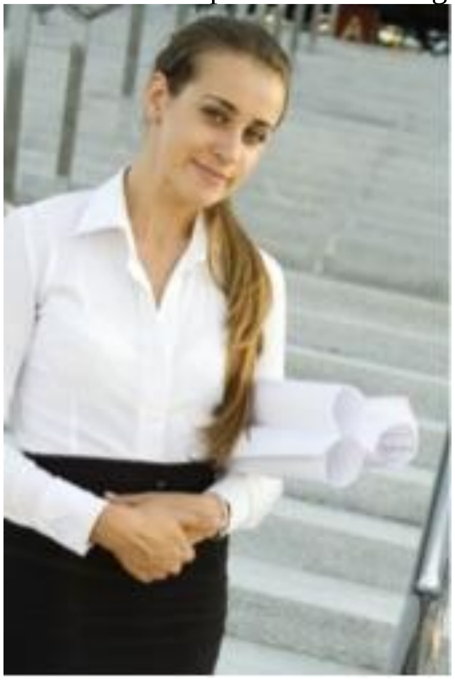

Maria

An engineering student named Maria wanted to do well in her program for the same reasons that everyone does - to get a good job, to meet her personal standards, to make her parents proud. But as a Hispanic woman, she spends every day being outnumbered by men in her engineering classes. Being a Hispanic woman puts her at risk of confirming the stereotype that women and Hispanics are not good at math and other quantitative skills. In addition to all the reasons she wants to do well in her courses, she has to deal with this extra pressure to perform well and to not conform to this stereotype. **All of this makes the engineering program a very difficult and stressful situation for Maria.**

Now we'd like you to think about what experiencing stereotype threat might be like. On the following pages you will read brief descriptions of people who are experiencing stereotype threat. We'd like you to imagine what these peoples' experiences might be like.

We'd like you to write a brief summary about the life of Katherine Parker, a student in an engineering program at an elite university.

For each of the scenarios below, please write 1 sentence about how Katherine might feel in these situations: As you do so, try to **feel** the full impact of Katherine's experiences and how she **feels** as a result.

Try to imagine how much more **difficult** her experiences are than the typical student, and how **stressful** or **challenging** it might be for her.

- Goes to a mechanical engineering lab, where she is the only female student in her lab group.

- Meets with her study group to prepare for an exam, and how she feels extra pressure to prove to the group that negative stereotypes about women's abilities in engineering aren't true.

- Meets with her Engineering professor, and how she might worry that the professor won't respect her abilities because she is a woman.

- Participates in an Engineering class discussion, and how she might worry that her classmates will judge her based on negative stereotypes about women in engineering.

- Takes a difficult Engineering exam, and how stressful and challenging the situation might be for her because of negative stereotypes about women's engineering abilities.

  Resilience focus condition.

<u>Article</u>

Please read the following article, taken from Claude Steele's book, *Whistling Vivaldi: And other*

clues to how stereotypes affect us. You will complete a short writing activity afterwards.

Consider the following thought experiment:

Imagine that you arrived late to your family reunion.

Thinking that everyone else has already eaten, you head to the buffet table and load your plate with food. But as you leave the buffet table, you notice a group of people who haven't eaten a bite yet. They look at the few remains on the food table and then at your plate filled with food. You try to explain, but your effort is lost in the noise of the crowd and the music. You hear them say as they go by, "Those Smiths are so selfish."

Based on this "misunderstanding" you might fear that your immediate family is going to be judged by the stereotype that they are selfish. Every time you do something that could be interpreted in light of that negative stereotype - such as volunteering to dry the dishes instead of wash, deciding how much to tip the pizza man - you are at risk of confirming their stereotype that your family is selfish. Such suspicion, and the unfair interpretation of your behavior, can make you feel uncomfortable. It could distract you and interfere with your interactions with other people. This experience is related to the experience of a threat from a negative stereotype - researchers call this "Stereotype threat."

But imagine how stereotype threat could get even worse. Suppose that the negative view of you had nothing to do with your own behavior but came from what people thought the group that you belong to, such as your race. Suppose that more people than just your relatives knew about this negative view - say everyone in society knew. Suppose that the negative views reflected not your table manners, but very important abilities that are important to getting ahead, for example, your intelligence in general. And, suppose that the new view applied to you in exactly those situations that were most critical to your success in school and in society - when you take a test, when you do a lab project. **Suppose you learn new strategies to perform your best in these situations despite the extra pressure created by stereotype threat.**

Stereotype threat describes the experience of being at risk of being judged through the lens of a negative stereotype. Everyone has different identities that have stereotypes attached to them - an older person might be judged by the stereotype that she is forgetful, a White man might be judged by the stereotype that white men can't dance, a person with glasses might be judged by the nerd stereotype.

When people have to perform while under the extra pressure of stereotype threat, it’s like when a track star runs a race against a headwind; their performance underestimates their true abilities. **People who experience stereotype threat have many strengths and skills that help them overcome the extra challenges created by stereotype threat.**

To see this in your own life, think of the important settings in your life: Your school, your workplace, your family. Imagine a situation in which your identity does matter in the situation, that it affects how you see things, whom you identity with, how you react emotionally to events

in the setting, and how you perform. **Think about the strategies you would use to manage the extra stress and pressure that the negative stereotype creates.**

Here is an example (a true story of a friend of the writer):

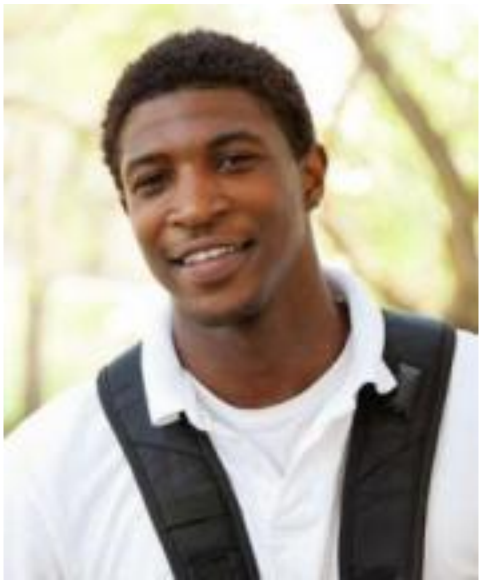

Jason

A college student named Jason was one of the only two Black students in an advanced writing class. In this context he worried about fitting in because he was Black, and he was concerned that he could be judged because of it - that if he said anything wrong or made any mistakes, he could confirm negative stereotypes about Black people. However, if he said nothing in class, then he could escape the suspicion of his fellow students. Because of these concerns, Jason experiences extra stress and anxiety in the class. His experiences are much more difficult than the average student. **He has developed and used the strengths he already had and has become a much stronger student as a result.**

Here is an example relevant to engineering (also a friend of the writer):

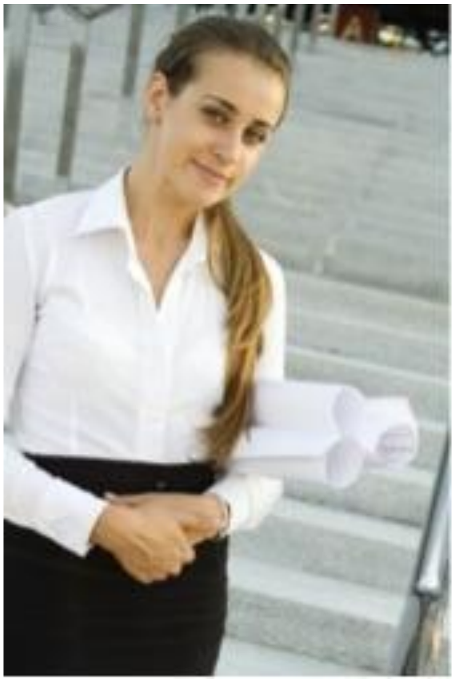

Maria

An engineering student named Maria wanted to do well in her program for the same reasons that everyone does - to get a good job, to meet her personal standards, to make her parents proud. But as a Hispanic woman, she spends every day being outnumbered by men in her engineering classes. Being a Hispanic woman puts her at risk of confirming the stereotype that women and Hispanics are not good at math and other quantitative skills. In addition to all the reasons she wants to do well in her courses, she has to deal with this extra pressure to perform well and to not conform to this stereotype. All of this makes the engineering program a very difficult and stressful situation for Maria. **She uses her strengths and skills to manage the extra stress and pressure to make her a better engineer.**

Now we'd like you to think about what experiencing stereotype threat might be like. On the following pages you will read brief descriptions of people who are experiencing stereotype threat. We'd like you to imagine what these peoples' experiences might be like.

We'd like you to write a brief summary about the life of Katherine Parker, a student in an engineering program at an elite university.

For each of the scenarios below, please write 1 sentence about how Katherine might feel in these situations: As you do so, try to **feel** the full impact of Katherine's experiences and how she **feels** as a result.

Try to imagine how she might **overcome the challenges she faces**, and how she uses her **strengths** and **skills t**o manage these difficult experiences.

- Goes to a mechanical engineering lab, where she is the only female student in her lab group.

- Meets with her study group to prepare for an exam, and how she has **learned** to **perform her best** despite the extra pressure to prove to the group that negative stereotypes about women's abilities in engineering aren't true.

- Meets with her Engineering professor, and what **strategies she uses to get the most out of the feedback** despite the professor's potential doubts about women's engineering abilities.

- Participates in an Engineering class discussion, and how she **demonstrates her engineering skills** to reduce her concerns that her classmates will judge her based on negative stereotypes about women in engineering.

- Takes a difficult Engineering exam, and what **strategies she uses** to reduce any concerns she has because of negative stereotypes about women's engineering abilities.

Study 5

Difficulties focus condition.

Now we'd like you to read about the life of Tyrone Williams, a student in his second year of college. Below are a series of scenarios that describe Tyrone's experiences. Please read each scenario carefully.

- Goes to a political science lecture, and he is the only Black student in the class.
- Meets with his English professor to talk about his term paper, and how he might worry that the professor might judge his abilities in light of negative stereotypes about Black people.
- Meets with a few other students to work on a group project for his biology class, and how he might worry that his classmates are judging his abilities based on negative stereotypes about Black people.
- Gives a presentation in history class, and how stressful or challenging the situation can be for him.
- Takes a difficult midterm test while under extra pressure to prove that the negative stereotype about Black people's abilities isn't true.

Resilience focus condition.

Now we'd like you to read about the life of Tyrone Williams, a student in his second year of college. Below are a series of scenarios that describe Tyrone's experiences. Please read each scenario carefully.

- Goes to a political science lecture, and he is the only Black student in the class.

- Meets with his English professor to talk about his term paper, and what strategies he uses to get the most out of the feedback despite the professor's potential doubts about his ability.
- Meets with a few other students to work on a group project for his biology class, and what he could teach them about working well together even when they have concerns about everyone working hard on the project.
- Gives a presentation in history class, and what he could teach other students about concerns about how others will perceive them in front of the class.
- Takes a difficult midterm test, and what strategies he has learned to use to reduce any anxiety he feels when taking this test.

Control focus condition.

Now we'd like you to read about the life of Tyrone Williams, a student in his second year of college. Below are a series of scenarios that describe Tyrone's experiences. Please read each scenario carefully.

- Goes to a political science lecture.
- Meets with his English professor to talk about his term paper.
- Meets with a few other students to work on a group project for his biology class.
- Gives a presentation in history class.
- Takes a difficult midterm test.

Study 3 target scenarios

<u>Writing Activity</u>

Now we'll like you to write a brief summary about the life of Tyrone Williams, a student in his second year of college.

Imagine a day in the life of this individual as if you were that person, looking at the world through his eyes and walking through the world in his shoes.

Please write 1 sentence for each scenario: As you do so try to feel the full impact of Tyrone's experiences and how he feels as a result.

- Goes to a political science lecture, and he is the only Black student in the class.

- Meets with his English professor to talk about his term paper, and is concerned about how the professor will judge his abilities.

- Meets with a few other students to work on a group project for his biology class, and wonders what these students think about his abilities.

- Takes a difficult midterm test on which he feels extra pressure because he's Black.

- Gives a presentation in history class in which he describes Black American's experience with segregation in the early 20th century.

Attrition rates for Studies 1-5

Study	Attrition rate by focus condition			Chi-Square	P-value
	Control	Difficulties	Resilience		
1	18.37%	28%	28.2%	1.66	0.437
2	8.62%	8.77%	6.56%	0.25	0.883
3	–	16.67%	24.28%	4.42	0.036
4	14.33%	15.85%	17.11%	1.01	0.604
5	16.4%	21.6%	20.0%	1.81	0.404

Note: In Study 4, attrition was slightly greater in the resilience focus condition relative to the control focus condition. Given that the difference was relatively small and that the results of Study 4 were consistent with the previous studies (as well as the additional studies reported below), we do not think that differential attrition is a plausible alternative explanation for the findings of Study 4.

Exact items from dependent measures used in Studies 1-4

Manipulation Check (Study 3 only).

1. How much do you think you were able to **empathize** with Tyrone? (i.e. to understand and share his feelings)
2. How much do you think you were able to remain **objective** and **detached** from Tyrone's feelings? (Reversed)

Items were rated on a 5-point scale, 1 = *not at all*; 2 = *not much;* 3 = *A moderate amount*; 4 = *very much*.

Helplessness and competence.

Instructions:

<u>**Impression Formation Task**</u>

Now, we are interested in your general impression of the individual you just wrote about.

Please rate the extent to which you think Tyrone Williams possesses the following traits.

- Victim
- Empowered
- Happy

- Uncertain
- Strong
- Intelligent
- Incapable
- Determined
- In need of help
- Weak
- Able to help others
- Needs support
- Has a lot to offer

- Items were rated on a 5-point scale (1 = *Not at all*; 2 = *Not much*; 3 = *A moderate amount*; 4 = *Very much*; 5 = *An extreme amount*).
- Helplessness composite: Victim, incapable, in need of help, weak, needs support.
- Competence composite: Empowered, strong, intelligent, determined, able to help others, has a lot to offer.
- Happy and uncertain were included as filler items and were not analyzed.

Exploratory measures used in Studies 1&3

Study 1.

Measures. Study 1 included the following more exploratory measures:

- Warmth. Warmth was measured as part of the "impression formation task" that was used to measure victimhood and competence. The items included:
  - Cold (reversed)
  - Warm
  - Sociable
- Modern racism (McConahay, 1986)
- Internal and external motivation to respond without prejudice (Plant & Devine, 1998).

Results. There was a significant effect of focus condition on warmth, $F(2, 142) = 8.63$, $p < 0.001$. Participants in the difficulties condition ($M = 3.18$, $SD = 0.69$) perceived the target as significantly less warm than did participants in the resilience condition ($M = 3.72$, $SD = 0.65$; $p < 0.001$) and the control condition ($M = 3.58$, $SD = 0.65$; $p = 0.013$). Participants in the resilience condition did not significantly differ from those in the control condition, $p = 0.54$. There were no significant effects of condition on any of the other dependent measures (i.e. IMS, EMS, Modern Racism; ps > 0.30).

Study 3.

Support for policies to help stigmatized groups.

How much would you support the following policies to help underrepresented racial minorities in college?

- A remedial program in which minority students are given easier material to help them catch up.

    - Academic advising to help minority students avoid classes in which they will experience stereotype threat.
    - An accelerated program where minority students are encouraged to take challenging courses.
    - A program in which minority students work in Faculty members' research labs.
    - A program in which minority students pursue independent study with a faculty mentor.
    - A mandatory summer bridge program in which minority students learn basic study skills.

- Items were rated on a 5-point scale (1 = *Not at all*; 2 = *Slightly*; 3 = *Moderately*; 4 = *Very much*; 5 = *Extremely*).
- There were no significant differences by condition on any of these items (*p*s >0.2)

Supplementary Note

Sample sizes differ across the studies in the main text. Study 1 has an N of 146, Study 2 has an N of 175. Then Study 3 has an N of 384, Study 4 has an N of 875, and Study 5 has an N of 605. The reason for the increased sample size beginning in Study 3 is that these studies were run over a long period of time, as research methodology in the field was rapidly evolving. By the time we designed Study 3 and began data collection, best practices were to run highly powered studies, so we increased our samples accordingly (in line with power calculations).

Supplementary Analyses

Study 4

Manipulation Check

The main text reports the main effect of empathy for this manipulation check. Here we report the full model. As illustrated in Figure S1, the pattern of the full model supports the conclusion that the empathy manipulation was effective.

There was no main effect of focus condition, $F(2, 869) = 0.023$, $p = 0.978$. Unexpectedly, an empathy by focus condition interaction emerged, $F(2, 869) = 8.11$, $p < 0.001$. The pattern of comparisons using Tukey's post hoc tests showed that the interaction emerged because the empathy manipulation was particularly effective for controls. Among participants in the no-empathy condition, controls ($M = 2.74$, $SD = .75$) reported even less empathy than the difficulties focus condition ($M = 2.95$, $SD = 0.65$, $p = .021$, $d = .30$) and showed a marginally significant trend in that direction compared to the resilience condition ($M = 2.91$, $SD = 0.65$, $p = 054$, $d= .24$). The difficulties and resilience conditions did not differ ($p = .946$, $d = .07$). Similarly, among participants in the empathy condition, controls ($M = 3.53$, $SD = .70$) reported even more empathy than the difficulties focus condition ($M = 3.32$, $SD = 0.69$, $p = .022$ $d = .30$) and a nonsignificant statistical trend relative to the resilience condition ($M = 3.35$, $SD = 0.62$, $p = .103$, $d = .28$). The difficulties and resilience focus conditions did not differ ($p = .829$, $d= .04$).

Figure S1. Marginal Means for Study 4 Manipulation Check

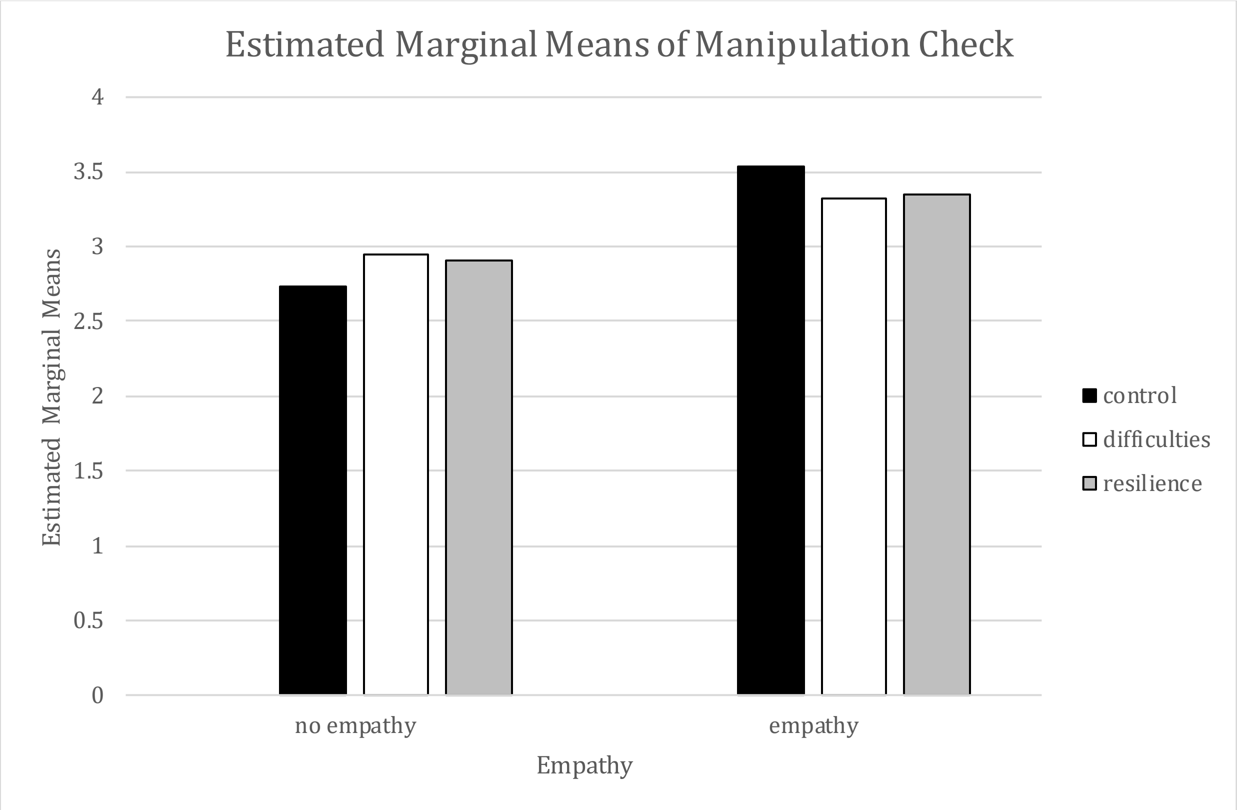

Supplementary studies

We ran several additional studies that were not included in the paper because of space constraints and the fact that the design and manipulations used in these studies were less clean than those reported in the paper, making their interpretation more difficult. The results across these studies were, for the most part, consistent with those reported in the paper.

Study S1

Overview

Study S1 was an initial exploratory pilot study examining the effects of the difficulties-focused approach vs. the resilience-focused approach on helplessness and competence, as well as a number of other exploratory dependent variables.

Method

Participants. Ninety-seven White Americans (59.8% female, M_{age} = 36.0, SD = 11.98) on MTurk participated in the study in exchange for payment.

Procedure. This study included three conditions: 1) a control focus condition with a White target; 2) a difficulties focus condition with a Black target; 3) a resilience focus condition with a Black target. As in Study 1 in the manuscript, in the two experimental conditions, participants first read about stereotype threat (modified from Steele, 2011; see below). Participants in the control condition read an article about plants. All participants then completed a perspective taking writing activity. In the two experimental conditions, this activity was identical to those used in Studies 1-3 (and is therefore not included below). The control condition for this study, however, included different scenarios and featured a White target (see below). As in Studies 1-3, participants then completed the measures of helplessness and competence, as well as number of exploratory measures.

Manipulation. The focus manipulation was identical to the one used in Study 1, with the exception of the perspective-taking writing exercise in the control condition, which is included below. In this study, the control perspective taking exercise featured a white target and scenarios that were unrelated to academics.

Control condition perspective taking exercise.

Now we'll like you to write a brief summary about the life of **John Williams**, a student in his second year of college.

Imagine a day in the life of this individual as if you were that person, **looking at the world through his eyes and walking through the world in his shoes.**

Please write 1 sentence for each scenario: As you do so try to **feel** the full impact of John's experiences and how he **feels** as a result.

- Wakes up and eats breakfast before class.
- Takes a nap.
- Takes his dog for a walk in the park.
- Goes to the grocery store.
- Watches a movie with his friends.

Measures. Study S1 included the measures of helplessness and competence used in Studies 1-3. In addition, Study S1 included the following more exploratory measures:

- Warmth. Warmth was measured as part of the "impression formation task" that was used to measure helplessness and competence. The items included:
    - Cold (reversed)
    - Warm
    - Sociable
- Modern racism (McConahay, 1986)

- Internal and external motivation to respond without prejudice (Plant & Devine, 1998).
- How confident are African Americans?
- How warm are African Americans?
- How well educated are African Americans?
- If African Americans get special breaks, this is likely to make things more difficult for people like me.
- How competent are African Americans?
- How economically successful are African Americans?
- How sincere are African Americans?
- Resources that go to African Americans are likely to take away from the resources of people like me.

Results

Helplessness and competence. There was a significant effect of focus condition on ratings of helplessness, $F(2, 93) = 17.21$, $p < 0.001$. Participants in the difficulties condition ($M = 2.48$, $SD = 0.74$) viewed the target as more helpless than did those in the control condition ($M = 1.68$, $SD = 0.48$; $p < 0.001$) and those in the resilience condition ($M = 1.86$, $SD = 0.46$; $p < 0.001$). Those in the resilience condition did not differ from those in the control ($p = 0.41$).

There was also a significant effect of focus condition on ratings of competence, $F(2, 93) = 10.32$, $p < 0.001$. Participants in the difficulties condition ($M = 3.64$, $SD = 0.77$) viewed the target as less competent than did those in the resilience condition ($M = 4.33$, $SD = 0.49$; $p < 0.001$) but they did not significantly differ from those in the control condition ($M = 3.72$, $SD = 0.68$; $p = 0.86$). Participants in the resilience condition rated the target as more competent than did those in the control condition ($p = 0.001$).

Warmth. There was a significant effect of focus condition on ratings of the target's warmth, $F(2, 93) = 11.03$, $p < 0.001$. Participants in the difficulties condition viewed the target as significantly less warm than did those in the control condition and the resilience condition (*p*s < 0.001). Those in the resilience condition did not significantly differ from those in the control ($p > 0.99$).

Internal and external motivation to respond without prejudice. There were significant effects of focus condition on internal motivation to respond without prejudice (IMS; $p = 0.001$) and on external motivation to respond without prejudice (EMS; $p = 0.042$). These effects, however, were not replicated in subsequent studies and so we do not discuss them further here.

Other measures. There were no significant differences by condition for any of the other variables listed above.

Study S2

Overview

The main goal of Study S2 was to examine the effects of the difficulties-focused approach vs. the resilience-focused approach across a broader set of dependent measures.

Method

Participants. One hundred and fifty-one White Americans on MTurk (63.6% female; $M_{age} = 38.52$; $SD = 12.50$) participated in the study in exchange for payment.

Procedures. The cover story, procedures, and materials of Study S2 were identical to Study 1 in the paper. Study S2, however, included several additional dependent measures that were not included in Study 1.

Measures. As in Study 1& S1, Study S2 included measures of helplessness, competence, warmth, IMS, EMS, and Modern Racism. In addition, Study S2 included the following measures:

- "How much do you like Tyrone?
- "If you were to meet Tyrone Williams, how would you feel towards him? **Please indicate how well each of the following words describes how you feel towards Tyrone."**
    - Positive
    - Negative
    - Sympathetic
    - Envious
    - Uneasy
    - Proud
    - Compassionate
    - Angry
    - Disgusted
    - Respectful
    - Pitying
    - Hateful
    - Frustrated
    - Jealous
    - Admiring
    - Resentful
    - Inspired
    - Contemptuous
    - Ashamed
    - Fond

Results

Helplessness and competence. There was a significant effect of focus condition on perceptions of helplessness, $F(2, 148) = 13.28$, $p < 0.001$. Participants in the difficulties condition ($M = 2.49$, $SD = 0.58$) perceived the target as more helpless than did those in the resilience condition ($M = 1.97$, $SD = 0.75$; $p < 0.001$) and the control condition ($M = 1.88$, $SD = 0.60$; $p < 0.001$). Participants in the resilience condition did not significantly differ from participants in the control condition, $p = 0.76$.

Focus condition also significantly affected perceptions of competence, $F(2, 148) = 4.62$, $p = 0.011$. Participants in the difficulties condition ($M = 3.82$, $SD = 0.62$) perceived the target as significantly less competent than did those in the resilience condition ($M = 4.21$, $SD = 0.65$; $p = 0.008$). Neither experimental condition significantly differed from the control condition, though both effects were in the predicted direction (ps > 0.1).

Warmth. Once again, there was a significant effect of focus condition on perceived warmth of the target, $F(2,148) = 5.94$, $p = 0.003$. Participants in the difficulties condition (M = 3.57, SD = 0.70) perceived the target as significantly less warm than did those in the resilience condition (M = 3.97, SD = 0.65; $p = 0.008$) and those in the control condition (M = 3.95, SD = 0.65; $p = 0.01$). Participants in the resilience condition did not significantly differ from those in the control condition, $p = 0.99$.

Other measures. There were significant and marginally significant effects of focus condition on a number of the more exploratory measures listed above. First, focus condition affected all three measures of overall evaluations of the target (i.e. liking, positivity, and negativity toward the target; ps < 0.05). Participants in the difficulties-focused condition liked the target less and felt less positively and more negatively about the target relative to participants in the control condition (ps < 0.05).

To create composites of participants' emotional reactions towards Tyrone, the items assessing specific emotional reactions were submitted to an exploratory factor analysis, which yielded four factors. The first factor included the following items: angry, uneasy, disgusted, hateful, frustrated, resentful, contemptuous, and ashamed; all of which involved negative emotional reactions to Tyrone. The second factor was made up of positive emotional reactions toward Tyrone and included the following items: proud, compassionate, respectful, admiring, inspired, and fond. The third factor involved feelings of concern for Tyrone and included sympathetic and pitying. The fourth and final factor included envious and jealous.

There were marginally significant effects of condition on the positive emotion composite and on the envy composite (ps < 0.10). Participants in the difficulties condition reported significantly less positive emotion toward Tyrone (vs. resilience; $p < 0.05$) and directionally less envy toward Tyrone (ps <0.20).

There were no significant effects of focus condition on any of the other measures of emotional reactions or on IMS/EMS.

Study S3

Overview

The goal of Study S3 was to test our hypotheses in a more controlled manner.

Method

Participants. Four hundred and forty-three White Americans (59.1% female, M_{age} = 39.75, SD = 13.59) on MTurk participated in the study in exchange for payment.

Procedures. The cover story, procedures, and materials were identical to Study 2 in the manuscript except for minor changes to the resilience condition. In particular, we revised the scenarios in the resilience condition so that they would be more consistent with those in the difficulties condition. The exact scenarios used in the resilience condition were:

- Goes to a political science lecture, and he is the only Black student in the class.

- Meets with his English professor to talk about his term paper, and how he might worry that the professor might judge his abilities in light of negative stereotypes about Black people, but how he might overcome these worries.

- Meets with a few other students to work on a group project for his biology class, and how he might worry that his classmates are judging his abilities based on negative stereotypes about Black people, but how he might handle these worries.

- Takes a difficult midterm test while under extra pressure to prove that the negative stereotype about Black people's abilities isn't true, and how he might cope with this extra pressure.

- Gives a presentation in history class, and how stressful or challenging the situation can be for him, but how he might overcome his stress.

Measures. Study S3 included only the focal dependent measures: helplessness and competence.

Results

Helplessness. There was a significant effect of condition on perceptions of helplessness, $F(2, 440) = 13.08$, $p < 0.001$. Participants in the difficulties condition ($M = 2.43$, $SD = 0.73$) perceived the target as significantly more helpless than did those in the control condition ($M = 2.02$, $SD = 0.65$; $p < 0.001$). Unlike the previous studies, however, participants in the difficulties condition did not significantly differ from those in the resilience condition ($M = 2.32$, $SD = 0.74$; $p = 0.36$). In addition, participants in the resilience condition perceived the target as significantly more helpless than did participants in the control condition, $p = 0.001$.

Competence. There was also a significant effect of condition on perceptions of competence, $F(2, 440) = 5.16$, $p = 0.006$. Participants in the difficulties condition ($M = 3.73$, $SD = 0.75$) perceived the target as significantly less competent than did those in the control condition (M = 3.98, SD = 0.58; $p = 0.005$) and marginally less competent than did those in the resilience condition ($M = 3.89$, $SD = 0.71$; $p = 0.095$). Participants in the resilience condition did not significantly differ from those in the control condition, $p = 0.54$.

Study S4

Overview

Study S4 was an exact replication of Study 3 in the main article. Study S4 was run before Study 3 and yielded trending, but non-significant results of the empathy manipulation. Thus, we ran Study 3 with double the sample size to see whether a significant effect of the empathy manipulation might emerge with more statistical power. For the sake of space, we chose to include only Study 3 (because it was more highly powered) in the article.

Method

Participants. Four hundred and fifty-one White Americans on MTurk (54.1% female, *M*age = 36.83, *SD* = 11.85) participated in the study in exchange for payment.

Procedure. All procedures and materials were identical to Study 3.

Results

Manipulation check. As expected, participants in the empathy condition reported significantly more empathy for the target than did participants in the no-empathy condition, $F(1, 445) = 64.05$, $p < 0.001$. Both the main effect of focus condition, as well as the empathy by focus condition interaction were non-significant (*p*s > 0.10).

Helplessness. As in Study 3, the empathy by focus condition interaction was nonsignificant, $F(2, 444) = 0.33$, $p = 0.72$. There was a trending effect of empathy condition on perceptions of helplessless such that participants in the empathy condition ($M = 2.25$, $SD = 0.68$) viewed the target as more helpless than did those in the no-empathy condition ($M = 2.15$, $SD = 0.71$); however, this effect was not statistically significant, $F(1, 444) = 2.71$; $p = 0.10$. In addition, there was a significant effect of focus condition on perceptions of helplessness, $F(2, 444) = 23.66$, $p < 0.001$. Participants in the difficulties condition ($M = 2.50$, $SD = 0.66$) viewed the target as significantly more helpless than did participants in the control condition ($M = 2.07$, $SD = 0.63$, $p < 0.001$) and the resilience condition ($M = 2.03$, $SD = 0.69$, $p < 0.001$). Participants in the resilience condition did not significantly differ from those in the control condition, $p = 0.82$.

Competence. The empathy by focus interaction was once again non-significant, $F(2, 444) = 1.73$, $p = 0.18$. In addition, the main effect of empathy condition was non-signicant, $F(1, 444) = 0.09$, $p = 0.76$. There was, however, a significant effect of focus condition, $F(2, 444) = 28.81$, $p < 0.001$. Participants in the difficulties condition ($M = 3.58$, $SD = 0.63$) viewed the target as significantly less competent than did participants in the resilience condition ($M = 4.09$, $SD = 0.62$; $p < 0.001$), however, they did not signficantly differ from those in the control condition ($M = 3.67$, $SD = 0.59$; $p = 0.37$). Participants in the resilience condition viewed the target as significantly more competent than did participants in the control condition, $p < 0.001$.

Study S5

The goal of Study S5 was to rule out differences in inferred school performance as a potential alternative explanation of the findings reported in Studies 1-3. [2]

Method

Participants. Three hundred White MTurk workers (55.7% female, $M_{age} = 37.08$, years, $SD = 12.25$) participated in the study in exchange for payment.

Procedure. The cover story, materials, procedures, and measures were identical to Study 2, except for the addition of information about the target's college performance in all three conditions. Specifically, prior to completing the empathy task, participants were given the following information about the target: "*Tyrone is a student in his second year of college. He attends a large flagship university in the Midwest. He is majoring in Political Science and his overall GPA is a 3.3.*"

[2] This study was run prior to Study 4 in the manuscript. Because Study 4 more definitively rules out potential alternative explanations associated with the content of the scenarios, including the one Study S5 was designed to address, we chose to include only Study 4 in the article.

Results. Once again, we found that focus condition significantly affected ratings of helplessness, $F(2, 297) = 14.85$, $p < 0.001$. Participants in the difficulties-focused condition perceived the target as more helpless ($M = 2.31$, $SD = 0.74$) than did those in the control condition ($M = 1.86$, $SD = 0.58$; $p < 0.001$) and the resilience condition ($M = 1.89$, $SD = 0.63$; $p < 0.001$. The control condition and the resilience condition did not significantly differ from one another, $p = 0.74$.

Focus condition also influenced participants' ratings of the target's competence, $F(2, 297) = 24.04$, $p < 0.001$. Participants in the difficulties condition ($M = 3.59$, $SD = 0.70$) rated the target as marginally less competent than did participants in the control condition ($M = 3.74$, $SD = 0.58$; $t(297) = -1.83$, $p = 0.069$) and significantly less competent than did those in the resilience condition ($M = 4.16$, $SD = 0.50$; $t(297) = -6.72$, $p < 0.001$). In contrast, participants in the resilience condition perceived the target as more competent than did those in the control condition, $t(297) = 4.90$, $p < 0.001$.

Other supplementary studies

We conducted two other supplementary studies that are not reported here for the sake of space. The first was an initial pilot test in which we crossed our perspective taking exercise with the stereotype threat article. Specifically, this study had a 2×2 design in which participants first read about either stereotype threat or the control article, and then completed the control perspective-taking activity or the resilience-focused perspective-taking activity. The second study was an initial follow-up to the research reported here in which we manipulated the target's race in addition to focus. This study is part of a separate and ongoing line of research and is thus not reported here. Data, materials, and results of these two studies are available upon request.

Discussion

Overall, the supplementary studies are consistent with the studies reported in the paper. In almost all of the studies described above, focusing on the target's difficulties caused participants to view the black target as more helpless and less competent. In contrast, focusing on the target's resilience led participants to view the target as more competent. We also found some evidence that these two approaches to stigma had effects on other outcomes, including warmth, evaluations of targets, and positive emotions; however, these findings were exploratory and should be treated with some degree of caution.

References

McConahay, J. (1986). Modern racism, ambivalence, and the Modern Racism Scale. In J. F. Dovidio and S. L. Gaertner (Eds.), *Prejudice, Discrimination, and Racism* (pp. 91–125). Orlando, FL: Academic Press.

McShane, B. B., & Böckenholt, U. (2017). Single-Paper Meta-Analysis: Benefits for Study Summary, Theory Testing, and Replicability. *Journal of Consumer Research*, *43*(6), 1048–1063. https://doi.org/10.1093/jcr/ucw085

Pigott, T. (2012). *Advances in Meta-Analysis*. New York: Springer-Verlag. Retrieved from //www.springer.com/us/book/9781461422778

Plant, E. A., & Devine, P. G. (1998). Internal and external motivation to respond without prejudice. *Journal of Personality and Social Psychology*, *75*(3), 811–832. https://doi.org/10.1037/0022-3514.75.3.811

Steele, C. M. (2011). *Whistling vivaldi: How stereotypes affect us and what we can do* (Reprint edition). New York: WW Norton.
